# Supplementary material for: Decisive role of water and protein dynamics in residence time of p38α MAP kinase inhibitors
Source: Nat Commun. 2022 Jan 28;13:569. doi: 10.1038/s41467-022-28164-4 (PMC8799644; doi:10.1038/s41467-022-28164-4)
Supplement: Supplementary file 1 — Supplementary Information [file 41467_2022_28164_MOESM1_ESM.pdf]

SUPPLEMENTARY INFORMATION FOR:

Decisive Role of Water and Protein Dynamics in Residence Time of p38α MAP Kinase Inhibitors

Tatu Pantsar[1,2], Philipp D. Kaiser[3], Mark Kudolo[1], Michael Forster[1], Ulrich Rothbauer[3,4,5], Stefan A. Laufer[1,5,6]*,

[1] Department of Pharmaceutical and Medicinal Chemistry, Institute of Pharmaceutical Sciences, Eberhard-Karls-Universität, Tübingen, Auf der Morgenstelle 8, 72076 Tuebingen, Germany.
[2] School of Pharmacy, Faculty of Health Sciences, University of Eastern Finland, Yliopistonranta 1, 70210 Kuopio, Finland.
[3] NMI Natural and Medical Sciences Institute at the University of Tuebingen, Markwiesenstrasse 55, Reutlingen, 72770, Germany.
[4] Pharmaceutical Biotechnology, Eberhard Karls University Tuebingen, Markwiesenstrasse 55, Reutlingen, 72770, Germany.
[5] Cluster of Excellence iFIT (EXC 2180) "Image-Guided and Functionally Instructed Tumor Therapies", University of Tuebingen, 72076 Tuebingen, Germany
[6] Tuebingen Center for Academic Drug Discovery & Development (TüCAD2), 72076 Tuebingen, Germany

* Correspondence: stefan.laufer@uni-tuebingen.de

Supplementary Figure 1. Cell-based eGFP-MK2 translocation assay: DLD1 cell line. 4

Supplementary Figure 2. Cell-based eGFP-MK2 translocation assay: HCT116 cell line. 5

Supplementary Figure 3. Ligands 1 and 2 are stable in the simulations. 6

Supplementary Figure 4. Comparison of the binding mode of 1 and 2. 7

Supplementary Figure 5. Structural evidence of disorder in crystal structure of 2 in complex with p38α MAPK and flexibility of G-loop. 8

Supplementary Figure 6. Selected lipophilic protein side chain contacts to compounds. 9

Supplementary Figure 7. Difference of metastable state 1-S_1 and 1-S_2 conformations near Thr175. 10

Supplementary Figure 8. Conformational discrepancy of metastable states 2-S_1, 2-S_2 and 2-S_3. 10

Supplementary Figure 9. Solvent exposure of the selected lipophilic residues. 11

Supplementary Figure 10. Solvent exposure of the side chains of Glu71 and Asp168. 12

Supplementary Figure 11. Hydration site displacement by 1. 13

Supplementary Figure 12. Hydration site displacement by 2. 14

Supplementary Figure 13. Well-tempered metadynamics simulations of 1-S_1 conformation I (20 replicates). 15

Supplementary Figure 14. Well-tempered metadynamics simulations of 1-S_1 conformation II (20 replicates). 16

Supplementary Figure 15. Well-tempered metadynamics simulations of 1-S_1 conformation III (20 replicates). 17

Supplementary Figure 16. Well-tempered metadynamics simulations of 1-S_2 conformation I (20 replicates). 18

Supplementary Figure 17. Well-tempered metadynamics simulations of 1-S_2 conformation II (20 replicates). 19

Supplementary Figure 18. Well-tempered metadynamics simulations of 1-S_2 conformation III (20 replicates). 20

Supplementary Figure 19. Well-tempered metadynamics simulations of 1-S_3 conformation I (20 replicates). 21

Supplementary Figure 20. Well-tempered metadynamics simulations of 1-S_3 conformation II (20 replicates). 22

Supplementary Figure 21. Well-tempered metadynamics simulations of 1-S_3 conformation III (20 replicates). 23

Supplementary Figure 22. Well-tempered metadynamics simulations of 2-S_1 conformation I (20 replicates).......24

Supplementary Figure 23. Well-tempered metadynamics simulations of 2-S_1 conformation II (20 replicates).......25

Supplementary Figure 24. Well-tempered metadynamics simulations of 2-S_1 conformation III (20 replicates).......26

Supplementary Figure 25. Well-tempered metadynamics simulations of 2-S_2 conformation I (20 replicates).......27

Supplementary Figure 26. Well-tempered metadynamics simulations of 2-S_2 conformation II (20 replicates).......28

Supplementary Figure 27. Well-tempered metadynamics simulations of 2-S_2 conformation III (20 replicates).......29

Supplementary Figure 28. Well-tempered metadynamics simulations of 2-S_3 conformation I (20 replicates).......30

Supplementary Figure 29. Well-tempered metadynamics simulations of 2-S_3 conformation II (20 replicates).......31

Supplementary Figure 30. Well-tempered metadynamics simulations of 2-S_3 conformation III (20 replicates).......32

Supplementary Figure 31. Key interactions observed in p38α MAPK–SB203580 complex simulations......33

Supplementary Figure 32. MSM metastable states of p38α MAPK–SB203580 complex.......34

Supplementary Figure 33. Hydration site displacement by SB203580......35

Supplementary Figure 34. Outline of the conducted simulations (1/2).......36

Supplementary Figure 35. Outline of the conducted simulations (2/2).......37

Supplementary Figure 36. Validation of Markov State Models of 1 and 2.......38

Supplementary Figure 37. Validation of Markov State Model of SB203580.......38

Supplementary Methods: Compound Synthesis......39

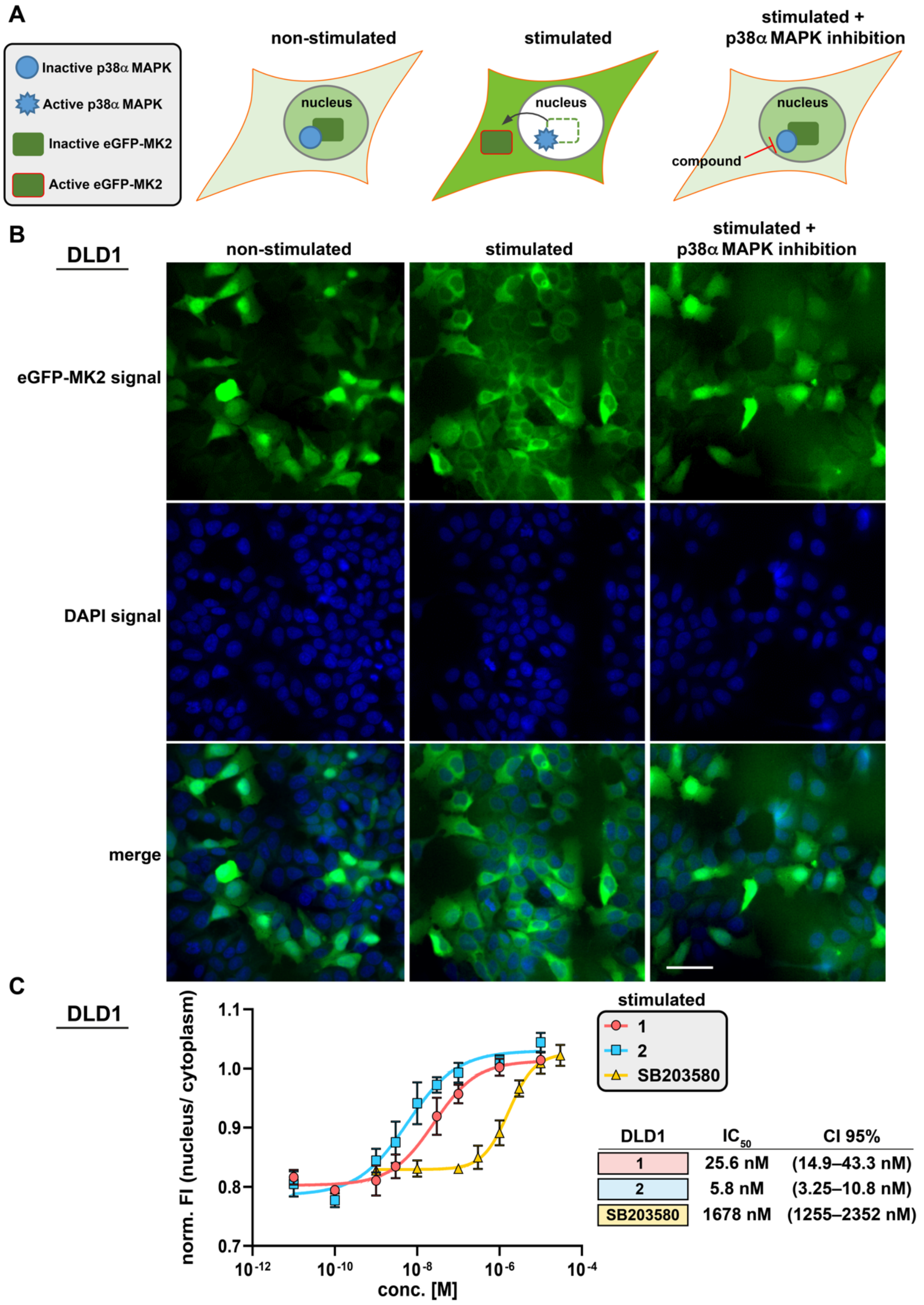

Supplementary Figure 1. **Cell-based eGFP-MK2 translocation assay: DLD1 cell line.** (**A**) Schematic overview of the cell-based eGFP-MK2 translocation assay. Active p38α MAPK phosphorylates its nuclear substrate MapKap2 (MK2) which leads to the exposure of the nuclear export signal and results in a cytoplasmic relocalization of MK2. Using a fluorescently labelled eGFP-MK2 as reporter, nuclear-cytoplasmic translocation can be used to monitor p38α MAPK activity upon compound induced modulation in live cells. (**B**) Human DLD1 cell line stably expressing eGFP-MK2 reporter under different treatment conditions. In non-stimulated cells, eGFP-MK2 signals predominantly localizes within the nucleus. Upon activation of the MAPK/p38

pathway, eGFP-MK2 is translocated to the cytoplasm which can be efficiently reverted by the addition of effective p38α MAPK inhibitors. Shown are microscopic images which were subjected to automated high content analysis to measure and calculate nuclear to cytoplasmic ratios of eGFP-MK2 signals providing the basis to evaluate inhibitory potency of p38α MAPK modulating compounds. Images are representative for 1 of 9 sites that were imaged per condition for each of 3 independent biological replicates. Scale bar: 50 µm. (**C**) Inhibition of eGFP-MK2 translocation by the compounds in DLD1_eGFP-MK2 cells. Normalized fluorescence (Fl) intensity ratio of nucleus/cytoplasm. IC_{50} values of 25.6 nM and 5.8 nM are observed for compound **1** and **2**, respectively. IC_{50} value of 1678 nM is observed for **SB203580**. Data are presented as mean ± standard deviation (SD) of three biological replicates. Source data are provided as a Source Data file.

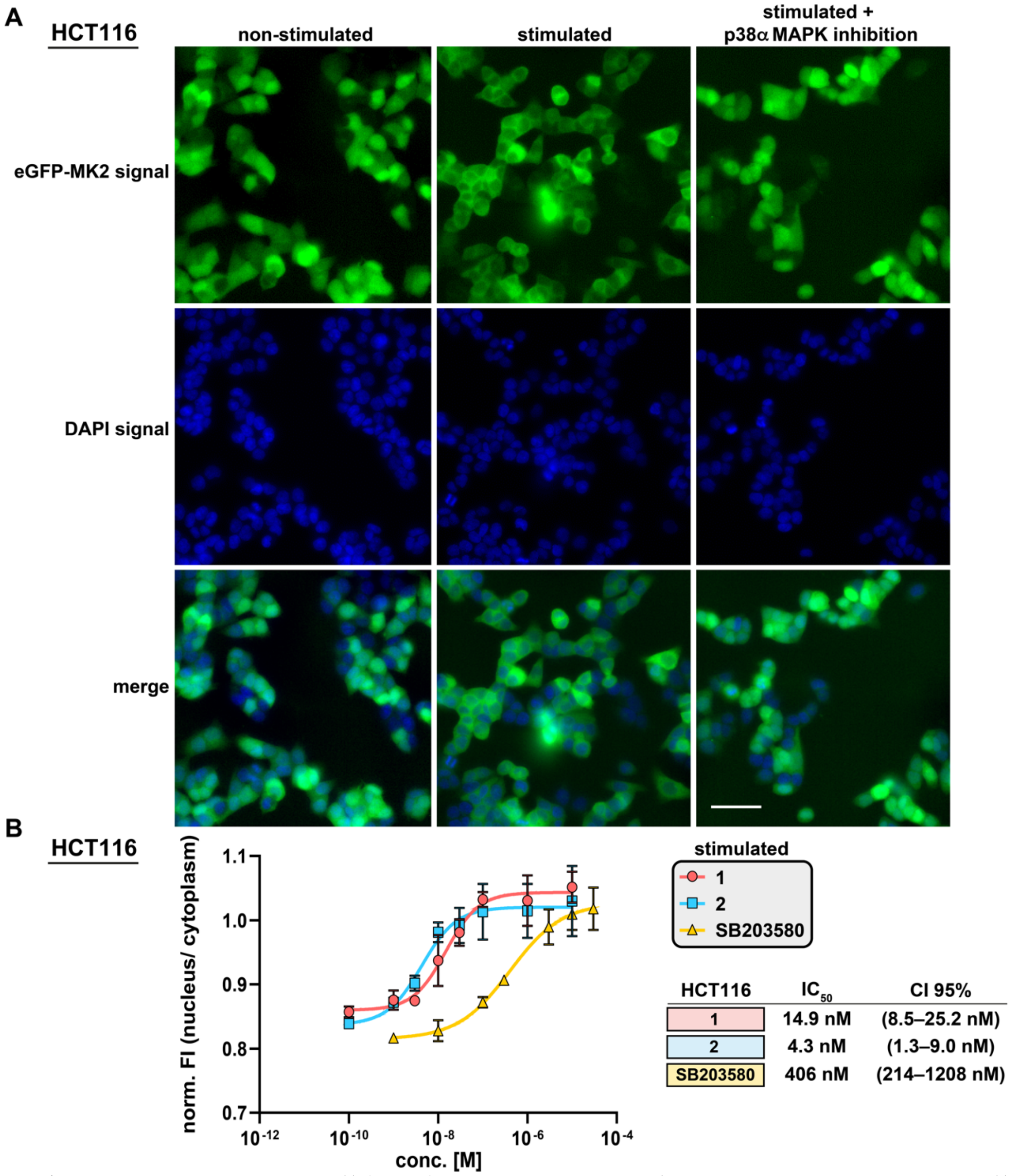

Supplementary Figure 2. **Cell-based eGFP-MK2 translocation assay: HCT116 cell line.** (**A**) Human HCT116 cell line stably expressing eGFP-MK2 reporter under different treatment conditions. In non-stimulated cells, eGFP-MK2 signals predominantly localizes within the

nucleus. Upon activation of the MAPK/p38 pathway, eGFP-MK2 is translocated to the cytoplasm which can be efficiently reverted by the addition of effective p38α MAPK inhibitors. Shown are microscopic images which were subjected to automated high content analysis to measure and calculate nuclear to cytoplasmic ratios of eGFP-MK2 signals providing the basis to evaluate inhibitory potency of p38α MAPK modulating compounds. Images are representative for 1 of 9 sites that were imaged per condition for each of 3 independent biological replicates. Scale bar: 50 µm. (**B**) Inhibition of eGFP-MK2 translocation by the compounds in HCT116_eGFP-MK2 cells. Normalized fluorescence (Fl) intensity ratio of nucleus/cytoplasm. IC_{50} values of 14.9 nM and 4.3 nM are observed for compound **1** and **2**, respectively. IC_{50} value of 406 nM is observed for **SB203580**. Data are presented as mean ± SD of three biological replicates. Source data are provided as a Source Data file.

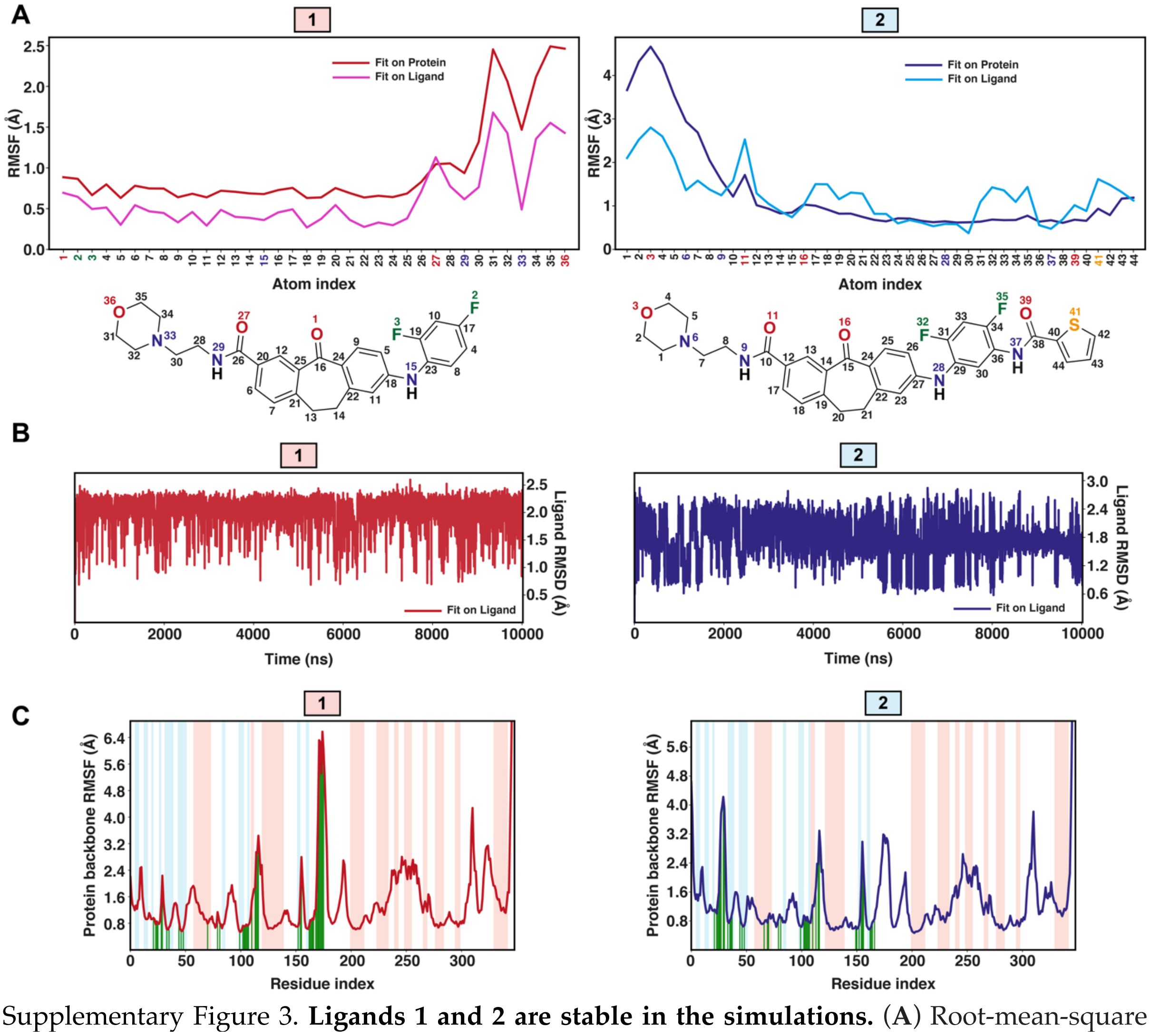

Supplementary Figure 3. **Ligands 1 and 2 are stable in the simulations.** (**A**) Root-mean-square fluctuation (RMSF) of **1** and **2** in the initial 10 µs simulations. Only the solvent exposed 2-morpholinoethylamide displays high-fluctuation during the simulations. Similar RMSF was observed also in the derived replica simulations (**B**) Root-mean-square deviation (RMSD) of **1** and **2** in the initial 10 µs simulations. (**C**) RMSF of protein backbone in the initial 10 µs simulations. Green vertical lines describe a ligand contact to the residue at some timepoint of the simulation. Secondary structure of the protein is described by blue and red shaded areas, which describe β-sheet and α-helix, respectively. Source data are provided as a Source Data file.

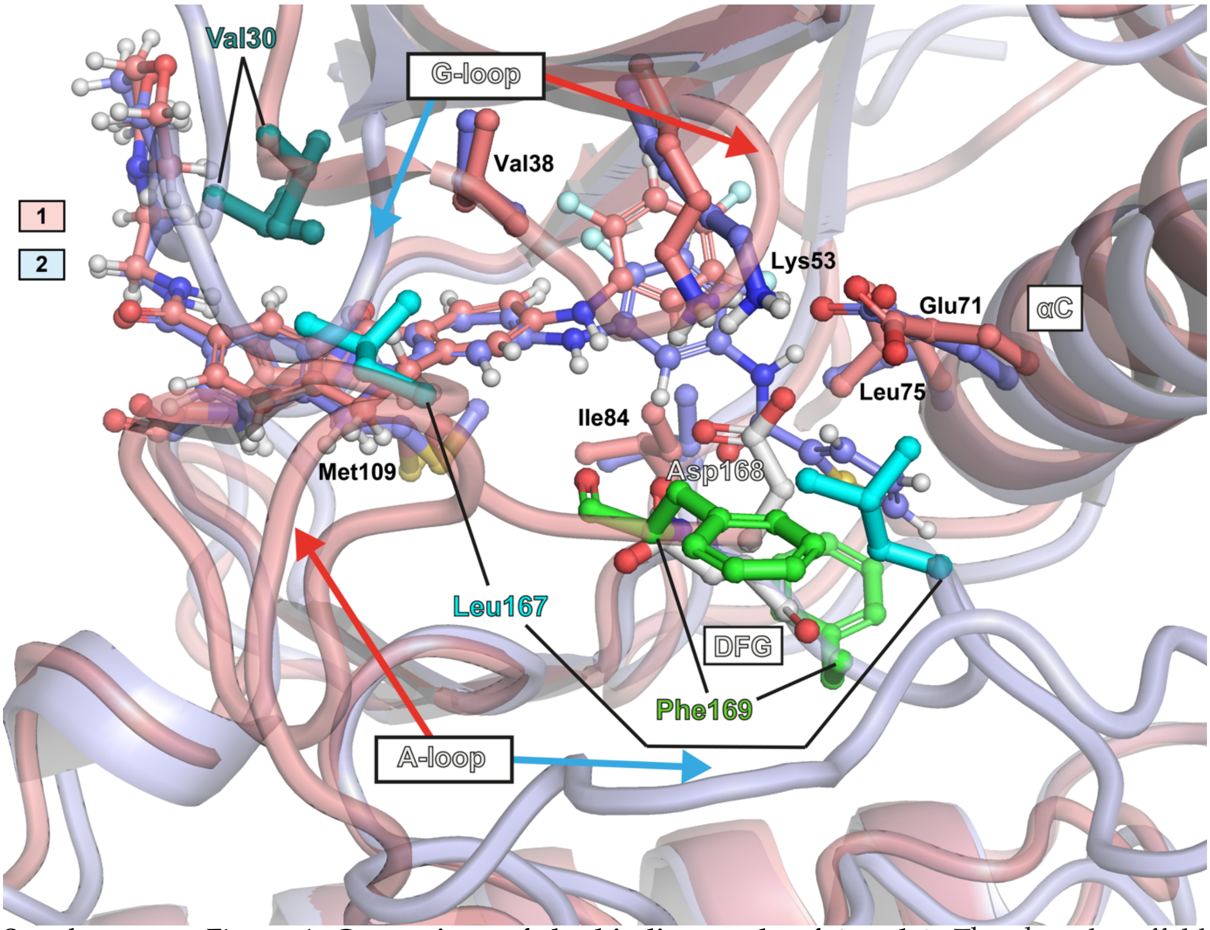

Supplementary Figure 4. **Comparison of the binding mode of 1 and 2.** The shared scaffold appears in similar position, with only a small shift in the difluorophenyl orientation. The different DFG orientation reflects also to the activation loop (A-loop) configuration (open/closed), and thereby to the positions of these residues (e.g. Leu167). The shifted G-loop orientation in **2** shifts the locations of the residues in this region. Other regions and their residues in the binding site appear in comparable positions.

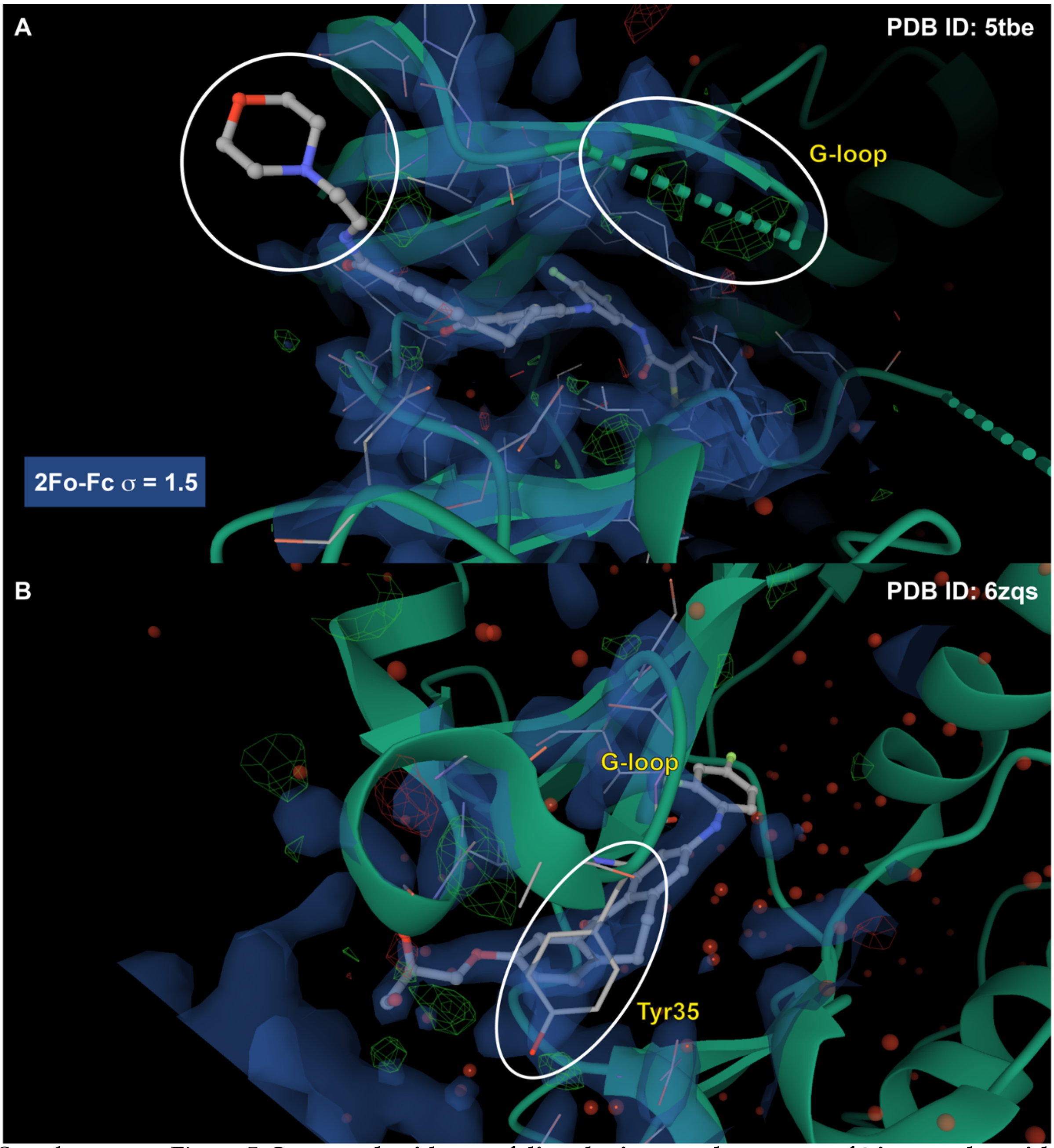

Supplementary Figure 5. **Structural evidence of disorder in crystal structure of 2 in complex with p38α MAPK and flexibility of G-loop.** (**A**) In crystal structure of **2** in complex with p38α MAPK, missing (delocalised) electron density is observed in G-loop and 2-morpholioethyl moiety. (**B**) In active conformation of MAPK p38α–Skepinone-L, dibenzosuberone moiety is shielded from solvent by Tyr35. 2Fo-Fc map contoured at 1.5 σ in both **A** and **B**.

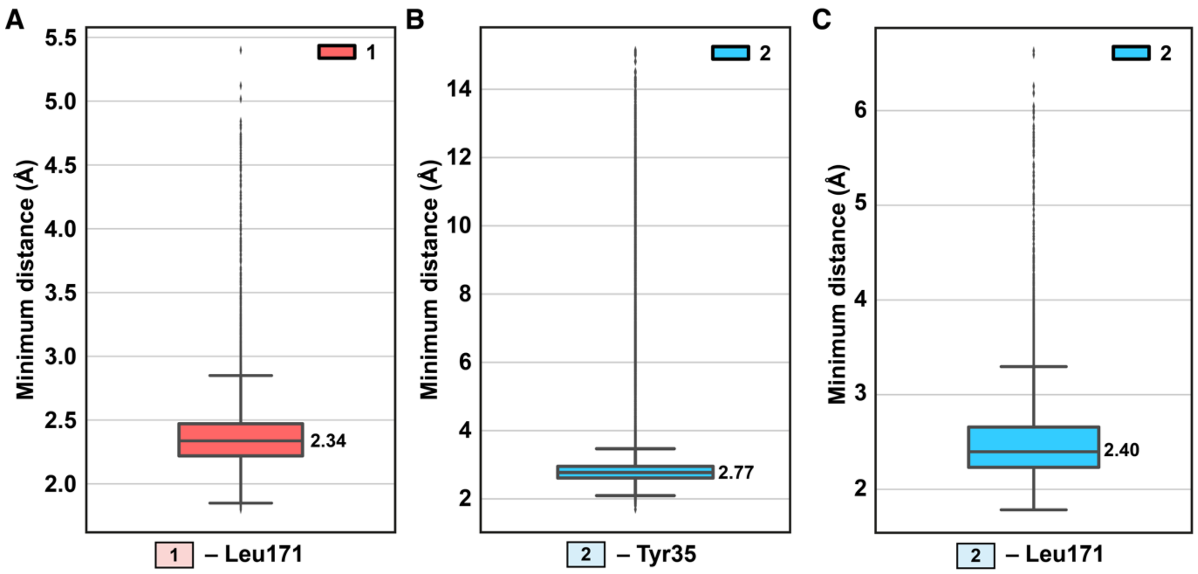

Supplementary Figure 6. **Selected lipophilic protein side chain contacts to compounds.** (**A**) Dibenzosuberone of **1** displays constant contact with side chain of Leu171, whereas (**B**) dibenzosuberone of **2** displays constant contact with side chain of Tyr35. (**C**) Leu171 displays contacts with the thiophene of **2**. The black horizontal line in the box represents the median. Box displays the quartiles of the dataset (25%–75%) and whiskers the rest of the data with maximum 1.5 IQR. Outliers are indicated with black diamonds. Simulation data was analysed for each 1 ns i.e. data consist of 91,328 and 86,505 individual data points for 1 and 2, respectively. Source data are provided as a Source Data file.

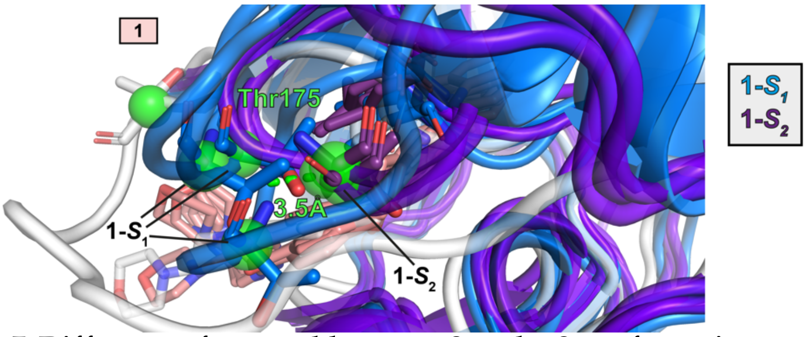

Supplementary Figure 7. **Difference of metastable state 1-S_1 and 1-S_2 conformations near Thr175.** An average distance difference of 3.5Å between Thr175 Cα-atoms is observed between these states.

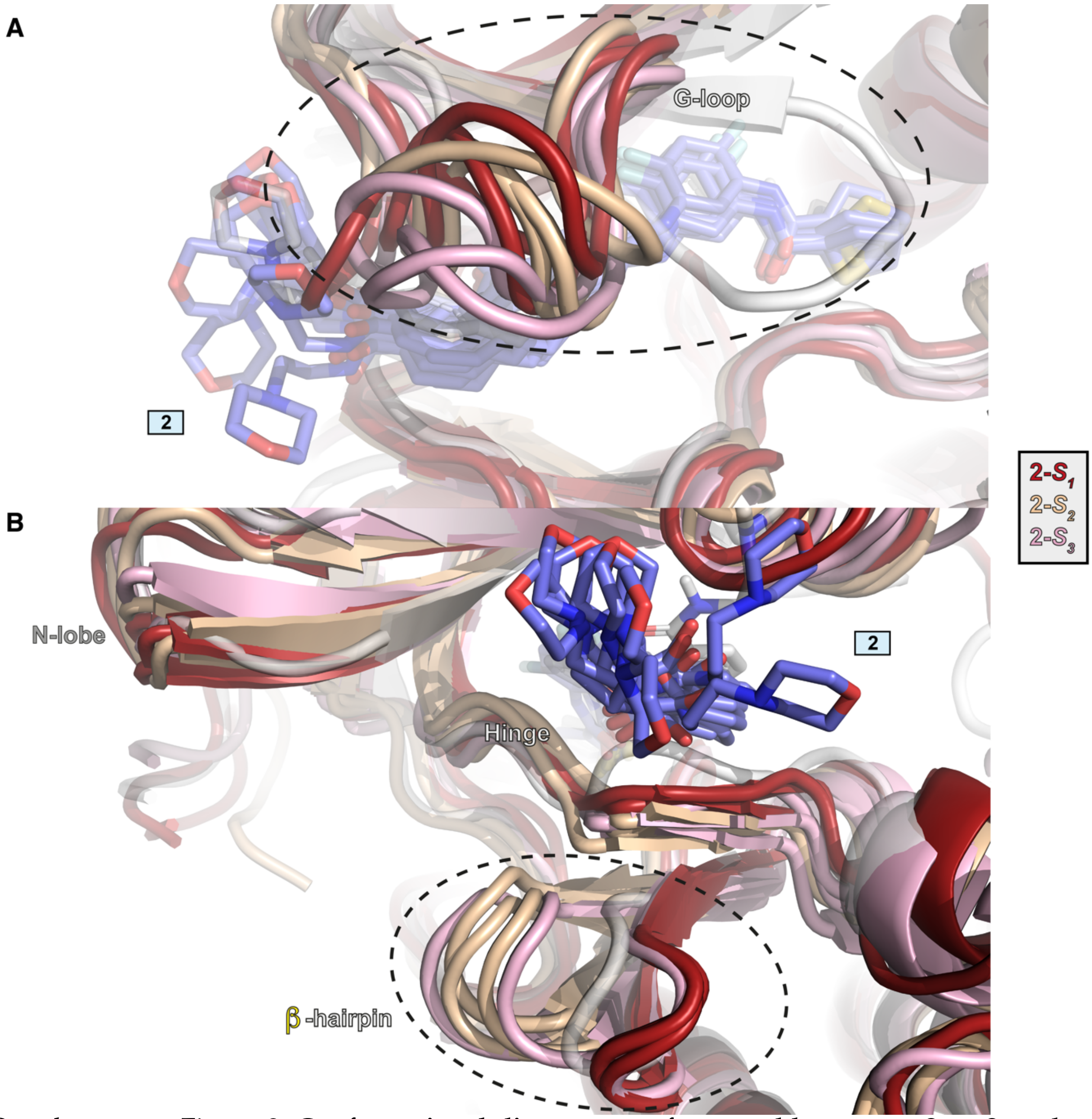

Supplementary Figure 8. **Conformational discrepancy of metastable states 2-S_1, 2-S_2 and 2-S_3.** Most notable conformational differences are observed in (**A**) G-loop and (**B**) β-hairpin near the hinge.

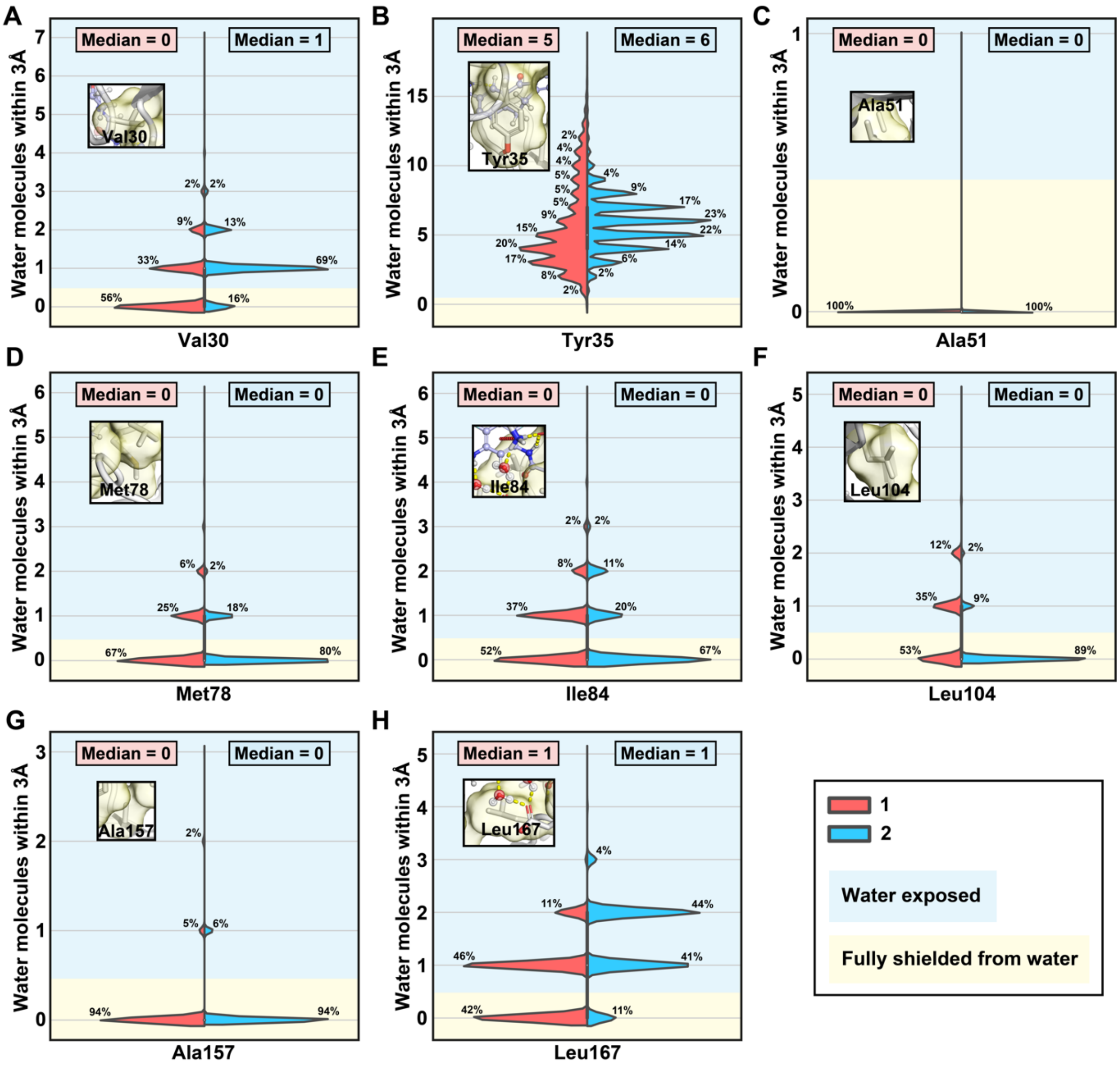

Supplementary Figure 9. **Solvent exposure of the selected lipophilic residues.** Solvent exposure of (**A**) Val30, (**B**) Tyr35, (**C**) Ala51, (**D**) Met78, (**E**) Ile84, (**F**) Leu104, (**G**) Ala157 and (**H**) Leu167 the blue background indicates solvent exposure (where >1 water molecule(s) is located within 3Å of the side chain) and the yellow background indicates when the side chain is not exposed to solvent. Source data are provided as a Source Data file.

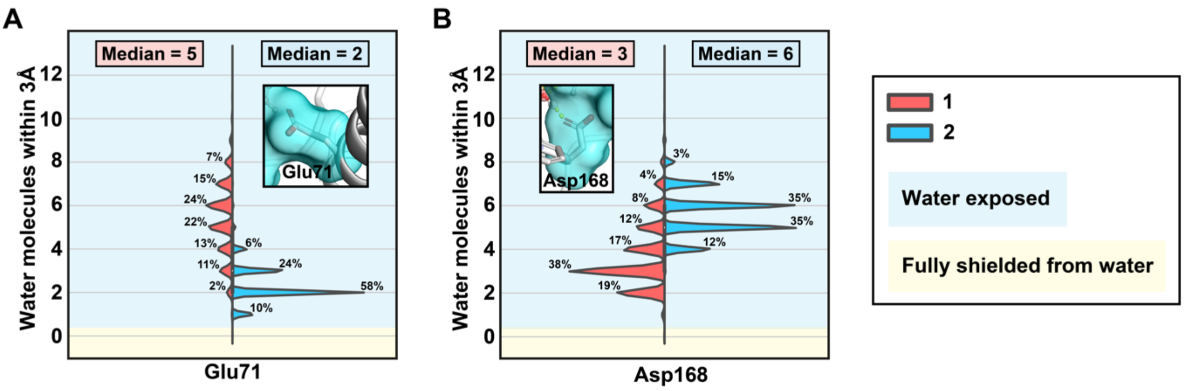

Supplementary Figure 10. **Solvent exposure of the side chains of Glu71 and Asp168.** Solvent exposure of (**A**) Glu71 and (**B**) Asp168 the blue background indicates solvent exposure (where >1 water molecule(s) is located within 3Å of the side chain) and the yellow background indicates when the side chain is not exposed to solvent. Source data are provided as a Source Data file.

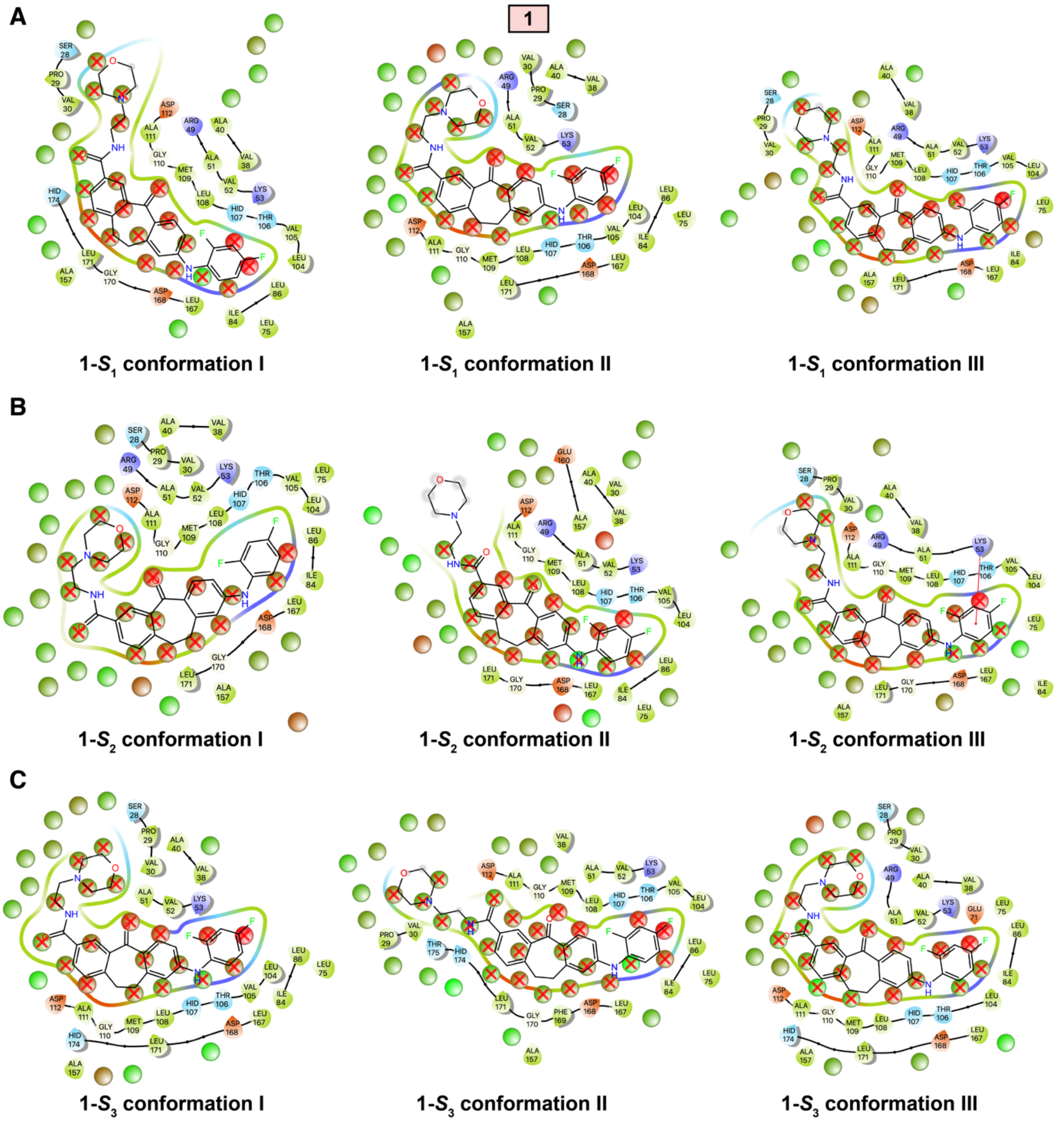

Supplementary Figure 11. **Hydration site displacement by 1.** 2D-represantation of the WaterMap results calculated for three conformations derived from (**A**) **1-*S*$_1$**, (**B**) **1-*S*$_2$** and (**C**) **1-*S*$_3$**. Hydration sites that would be displaced by **1** are marked with red cross mark.

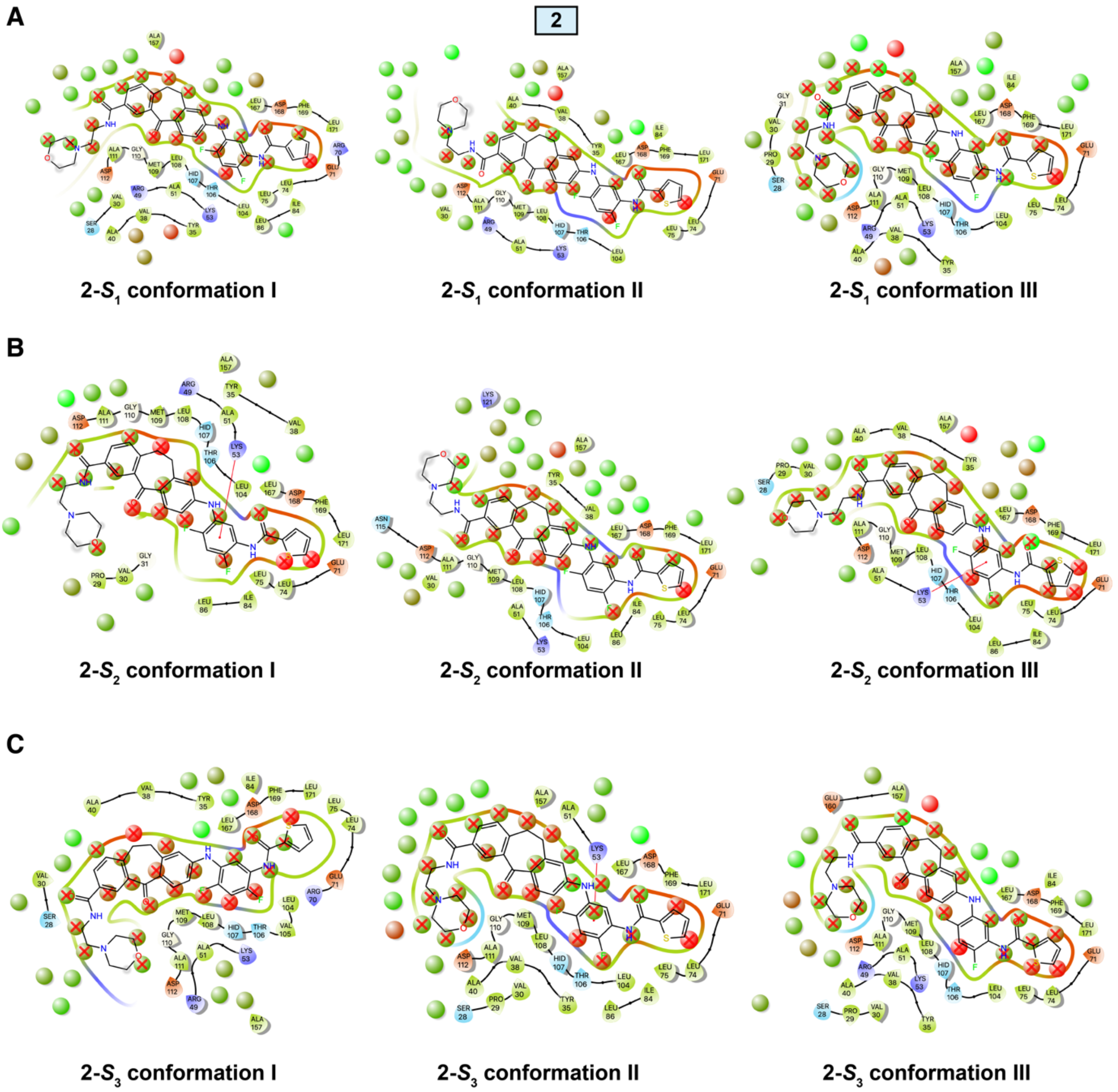

Supplementary Figure 12. **Hydration site displacement by 2.** 2D-represantation of the WaterMap results calculated for three conformations derived from (**A**) **2-*S*$_1$**, (**B**) **2-*S*$_2$** and (**C**) **2-*S*$_3$**. Hydration sites that would be displaced by **2** are marked with red cross mark.

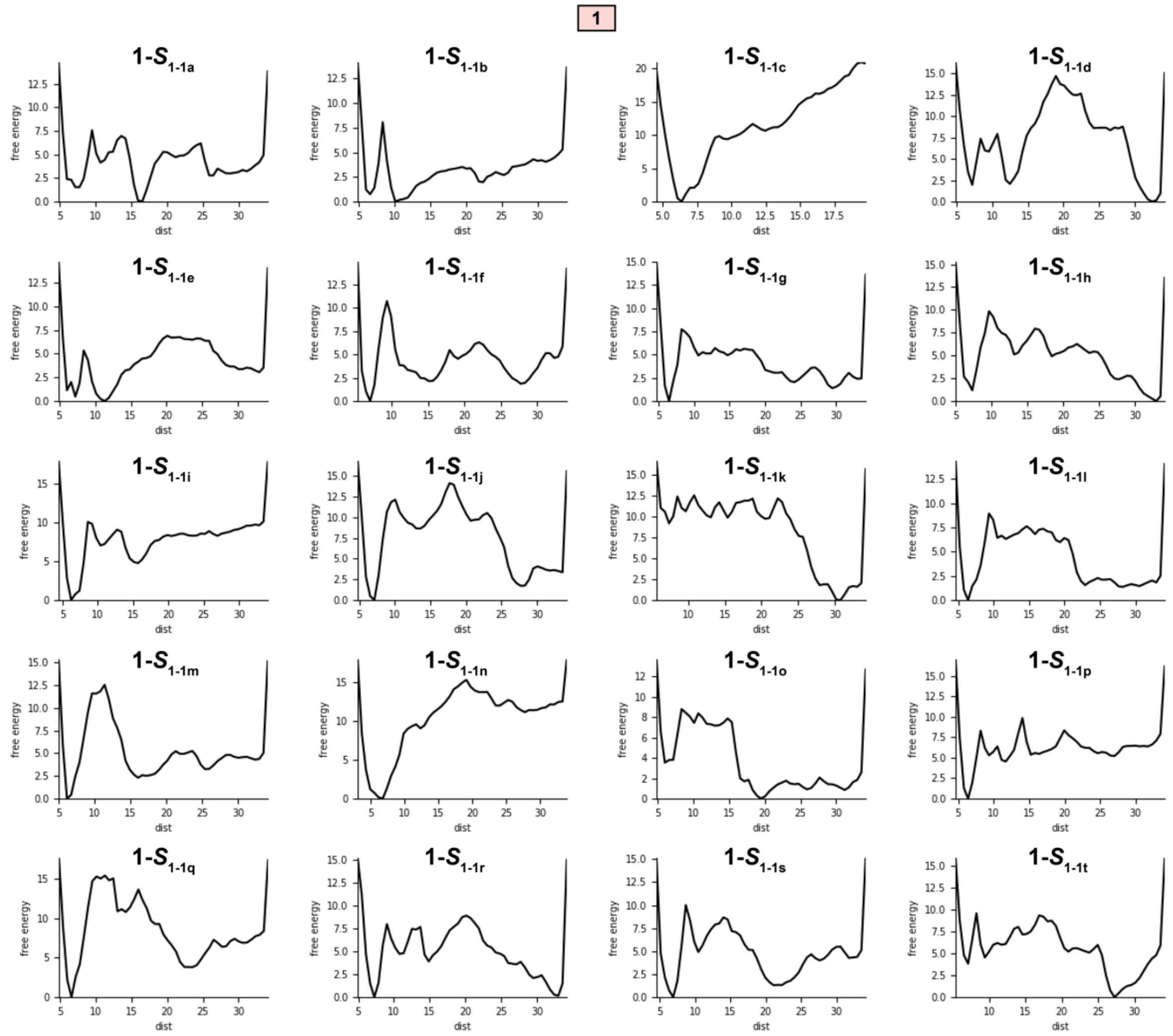

Supplementary Figure 13. **Well-tempered metadynamics simulations of 1-S_1 conformation I (20 replicates).** In the y-axis free energy and in the x-axis distance between the centre of mass of binding site residues and centre of mass of the inhibitor. Each simulation is 200 ns.

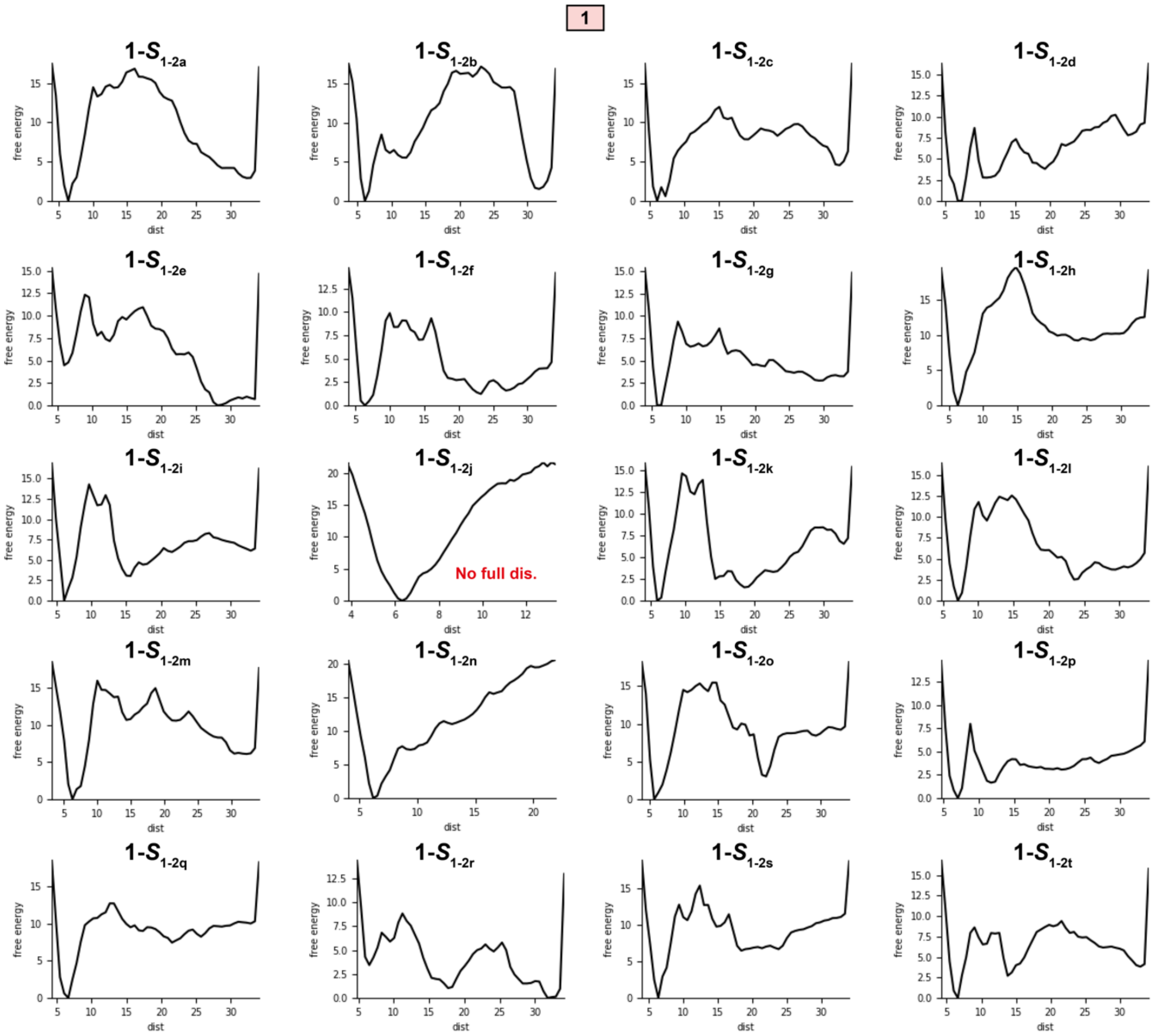

Supplementary Figure 14. **Well-tempered metadynamics simulations of 1-S_1 conformation II (20 replicates).** In the y-axis free energy and in the x-axis distance between the centre of mass of binding site residues and centre of mass of the inhibitor. Each simulation is 200 ns.

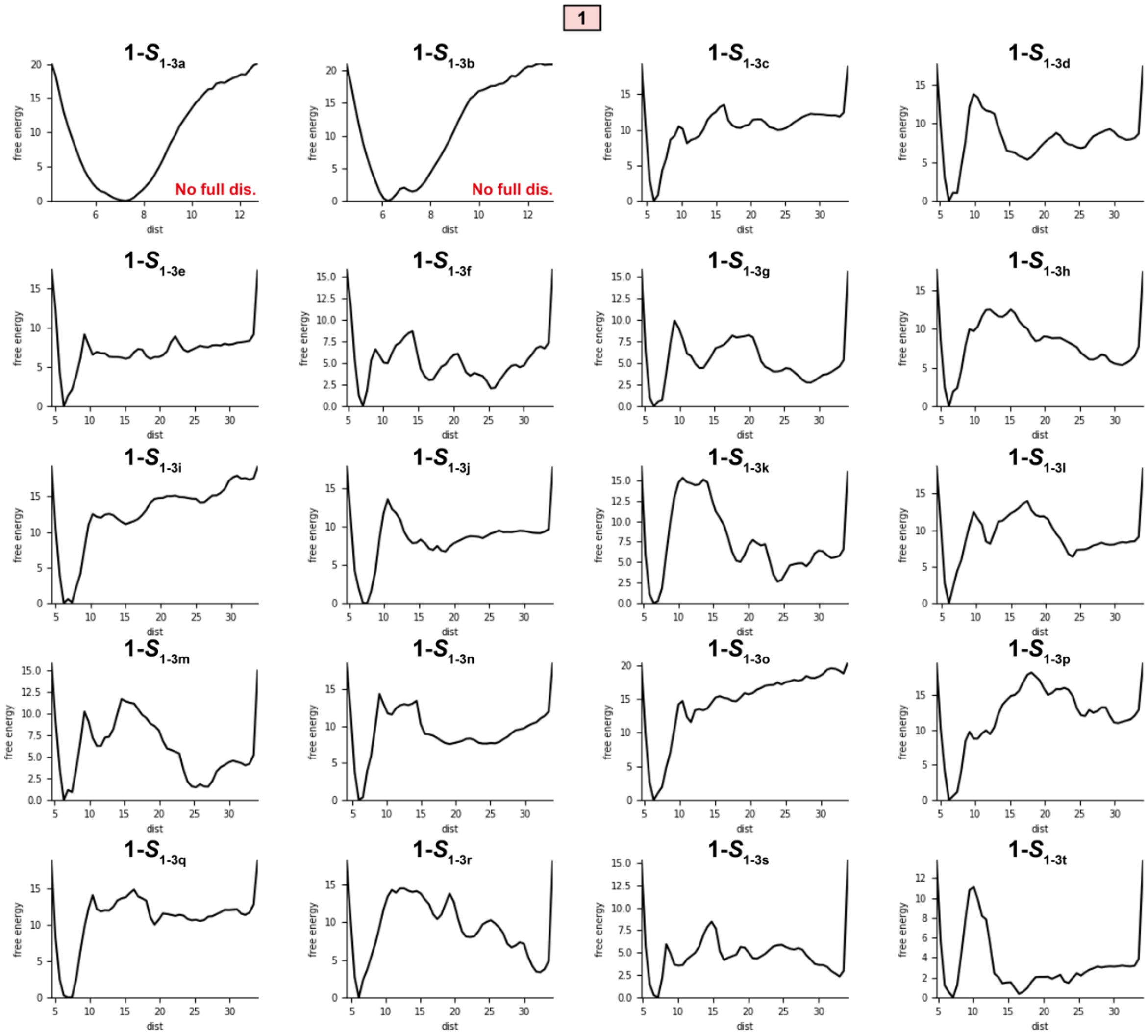

Supplementary Figure 15. **Well-tempered metadynamics simulations of 1-*S*$_1$ conformation III (20 replicates).** In the y-axis free energy and in the x-axis distance between the centre of mass of binding site residues and centre of mass of the inhibitor. Each simulation is 200 ns.

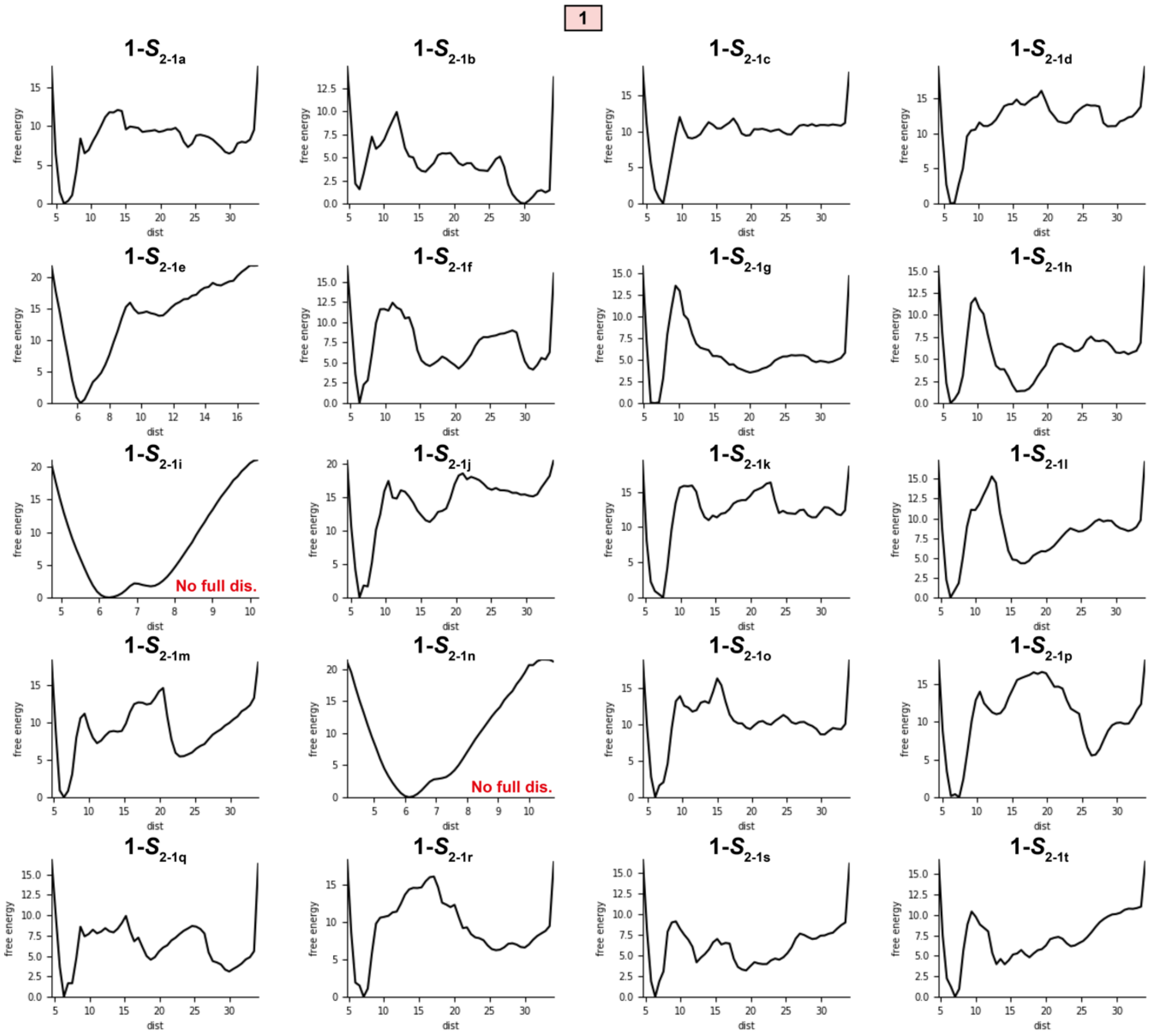

Supplementary Figure 16. **Well-tempered metadynamics simulations of 1-*S*$_2$ conformation I (20 replicates).** In the y-axis free energy and in the x-axis distance between the centre of mass of binding site residues and centre of mass of the inhibitor. Each simulation is 200 ns.

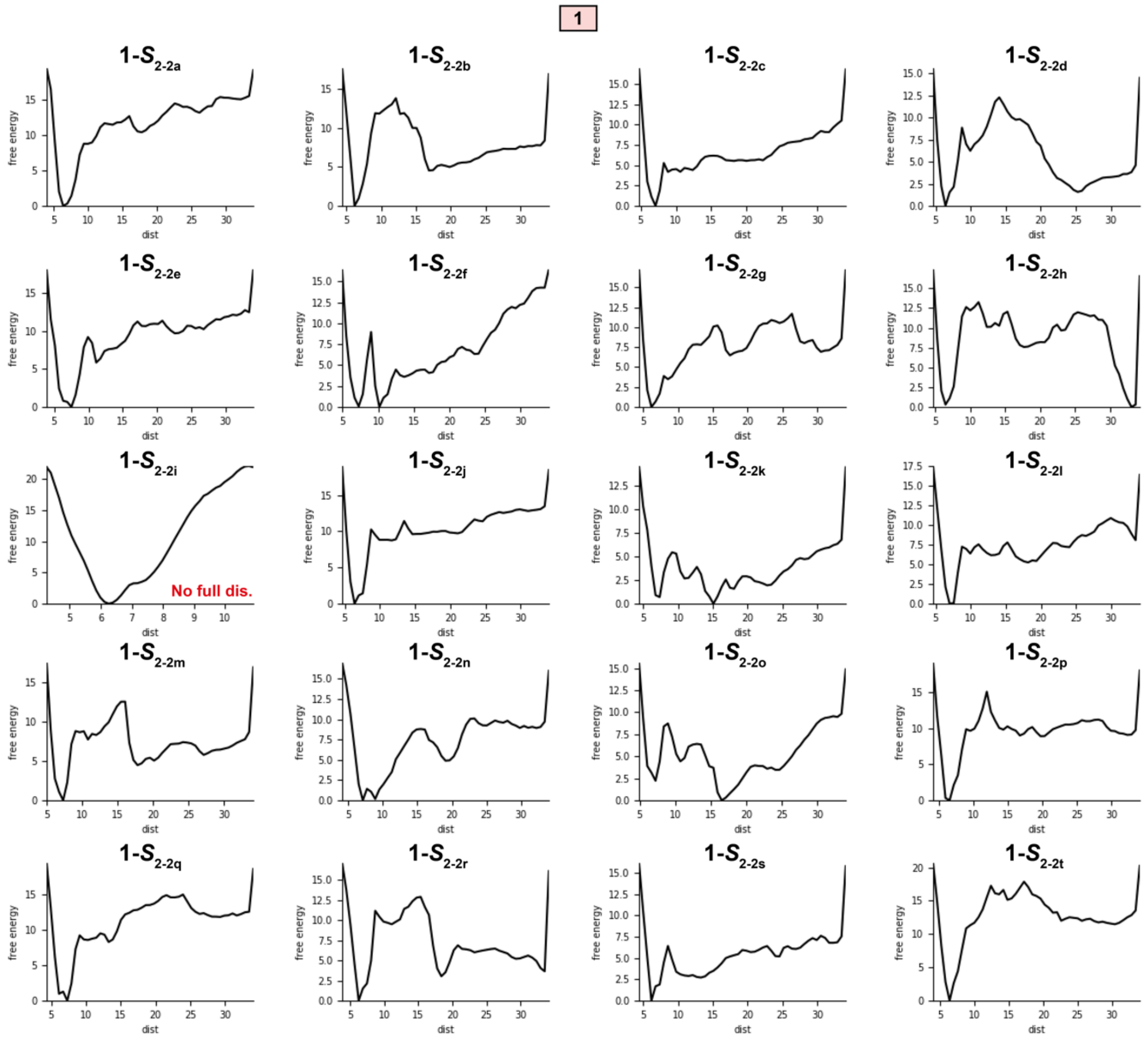

Supplementary Figure 17. **Well-tempered metadynamics simulations of 1-S_2 conformation II (20 replicates).** In the y-axis free energy and in the x-axis distance between the centre of mass of binding site residues and centre of mass of the inhibitor. Each simulation is 200 ns.

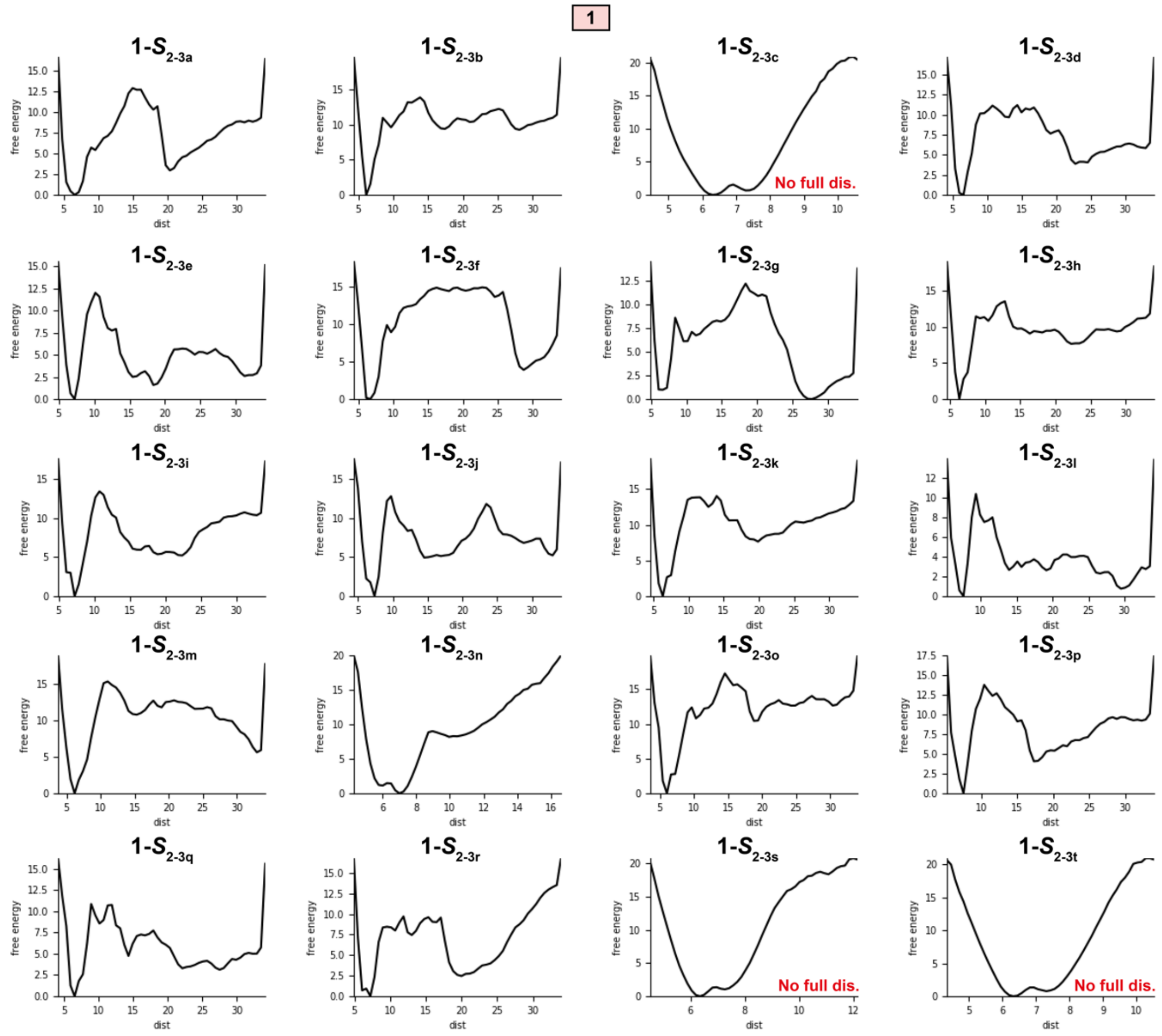

Supplementary Figure 18. **Well-tempered metadynamics simulations of 1-*S*$_2$ conformation III (20 replicates).** In the y-axis free energy and in the x-axis distance between the centre of mass of binding site residues and centre of mass of the inhibitor. Each simulation is 200 ns.

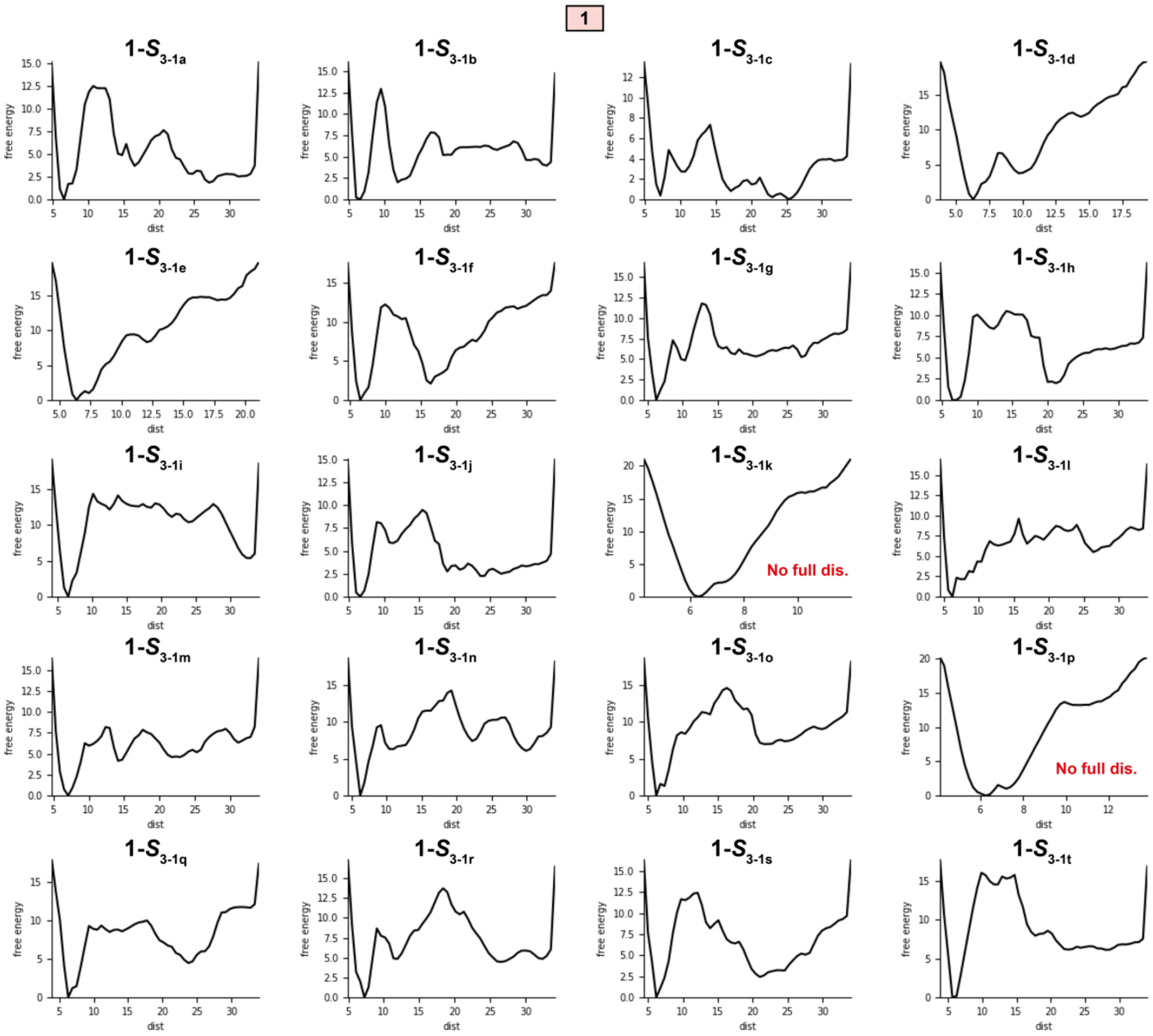

Supplementary Figure 19. **Well-tempered metadynamics simulations of 1-S_3 conformation I (20 replicates).** In the y-axis free energy and in the x-axis distance between the centre of mass of binding site residues and centre of mass of the inhibitor. Each simulation is 200 ns.

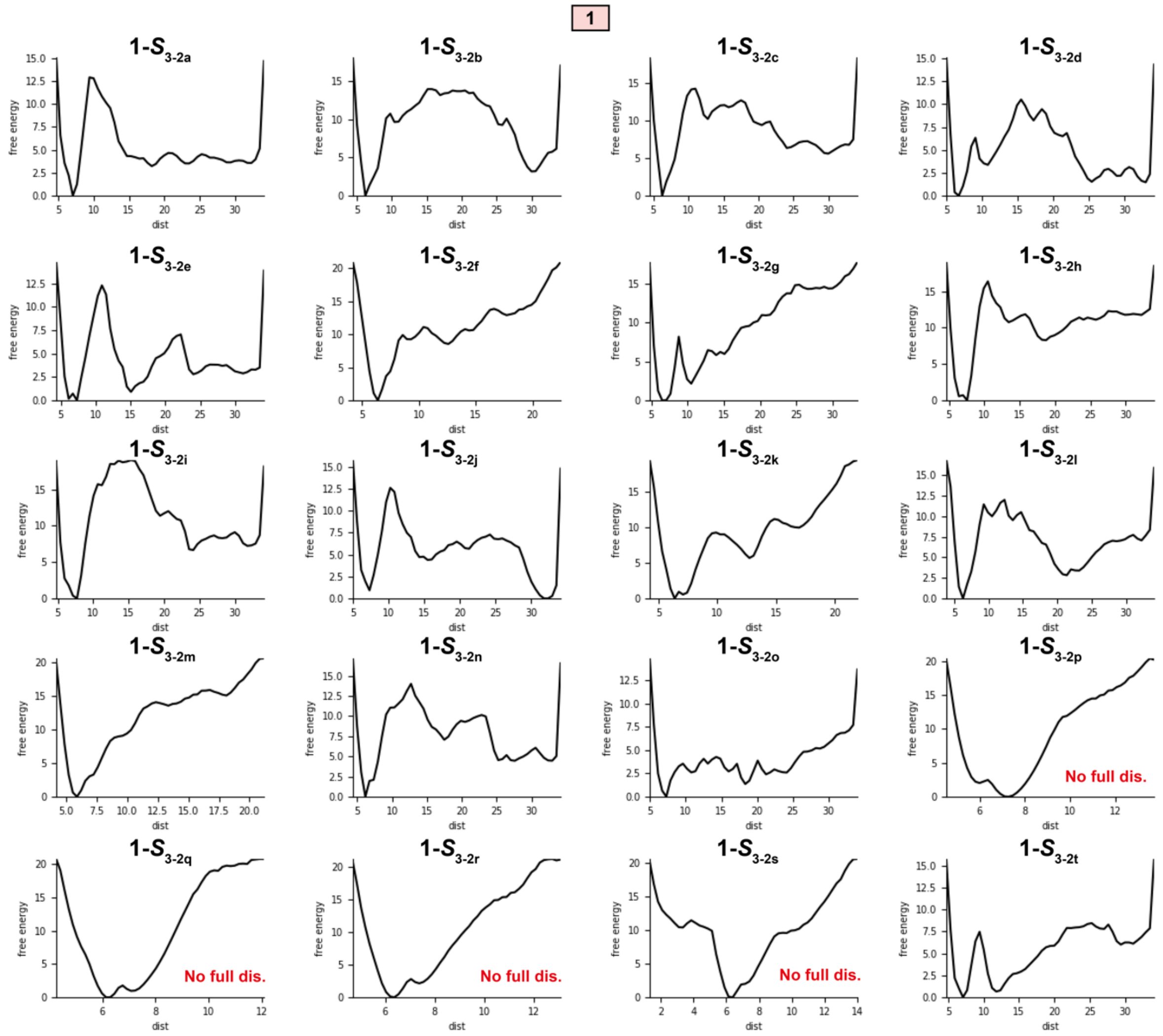

Supplementary Figure 20. **Well-tempered metadynamics simulations of 1-*S*$_3$ conformation II (20 replicates).** In the y-axis free energy and in the x-axis distance between the centre of mass of binding site residues and centre of mass of the inhibitor. Each simulation is 200 ns.

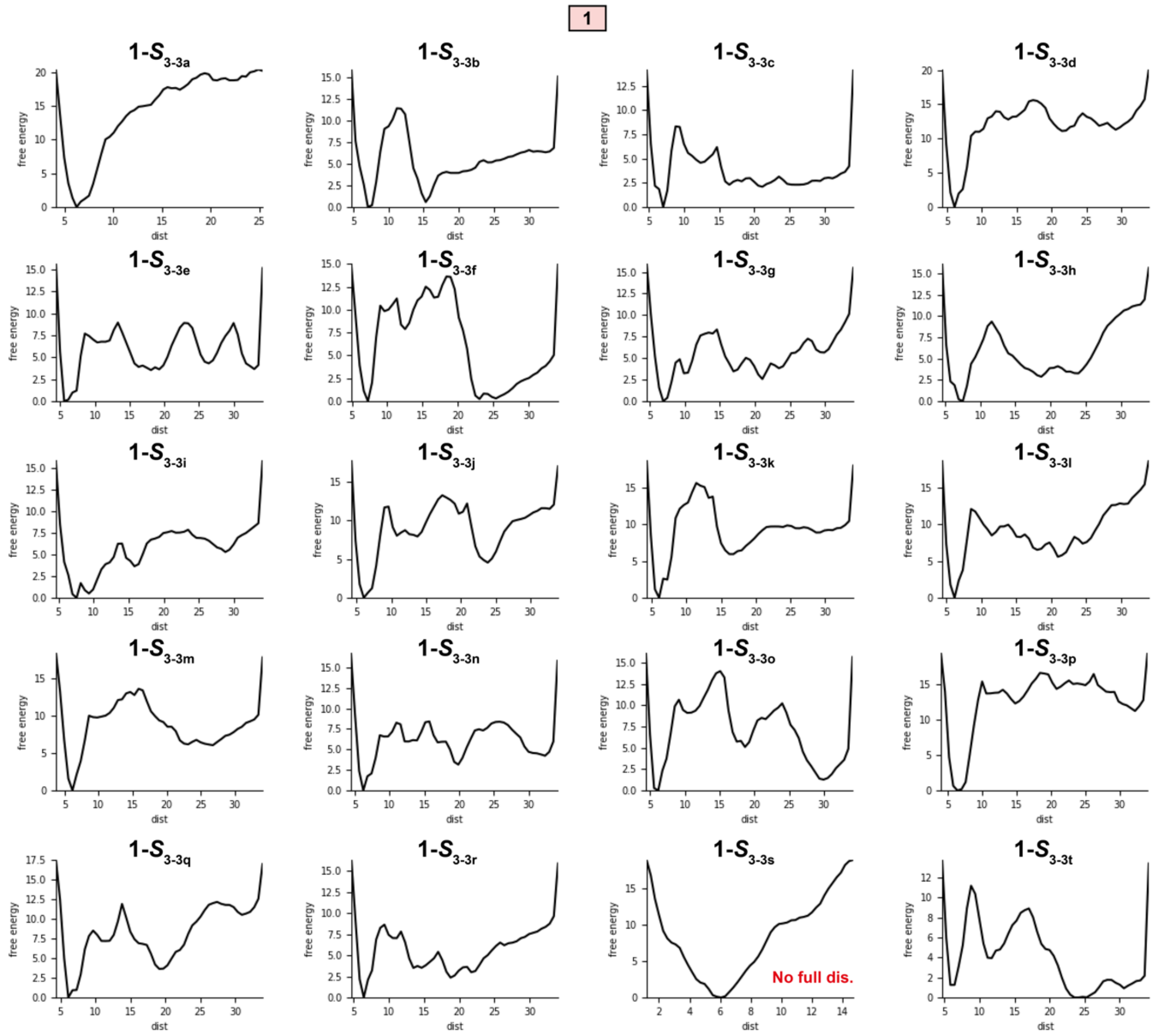

Supplementary Figure 21. **Well-tempered metadynamics simulations of 1-S_3 conformation III (20 replicates).** In the y-axis free energy and in the x-axis distance between the centre of mass of binding site residues and centre of mass of the inhibitor. Each simulation is 200 ns.

2

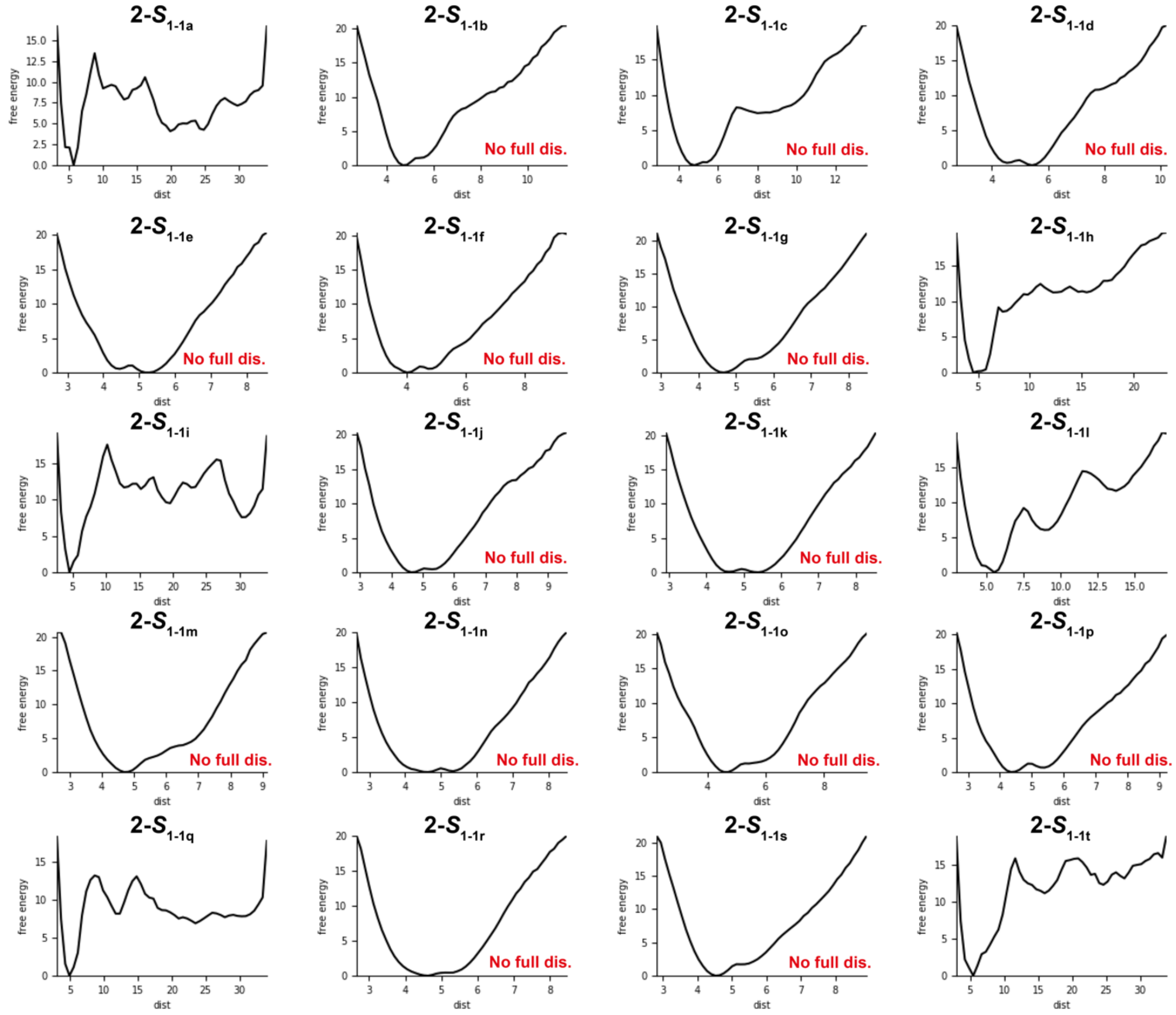

Supplementary Figure 22. **Well-tempered metadynamics simulations of 2-S_1 conformation I (20 replicates).** In the y-axis free energy and in the x-axis distance between the centre of mass of binding site residues and centre of mass of the inhibitor. Each simulation is 200 ns.

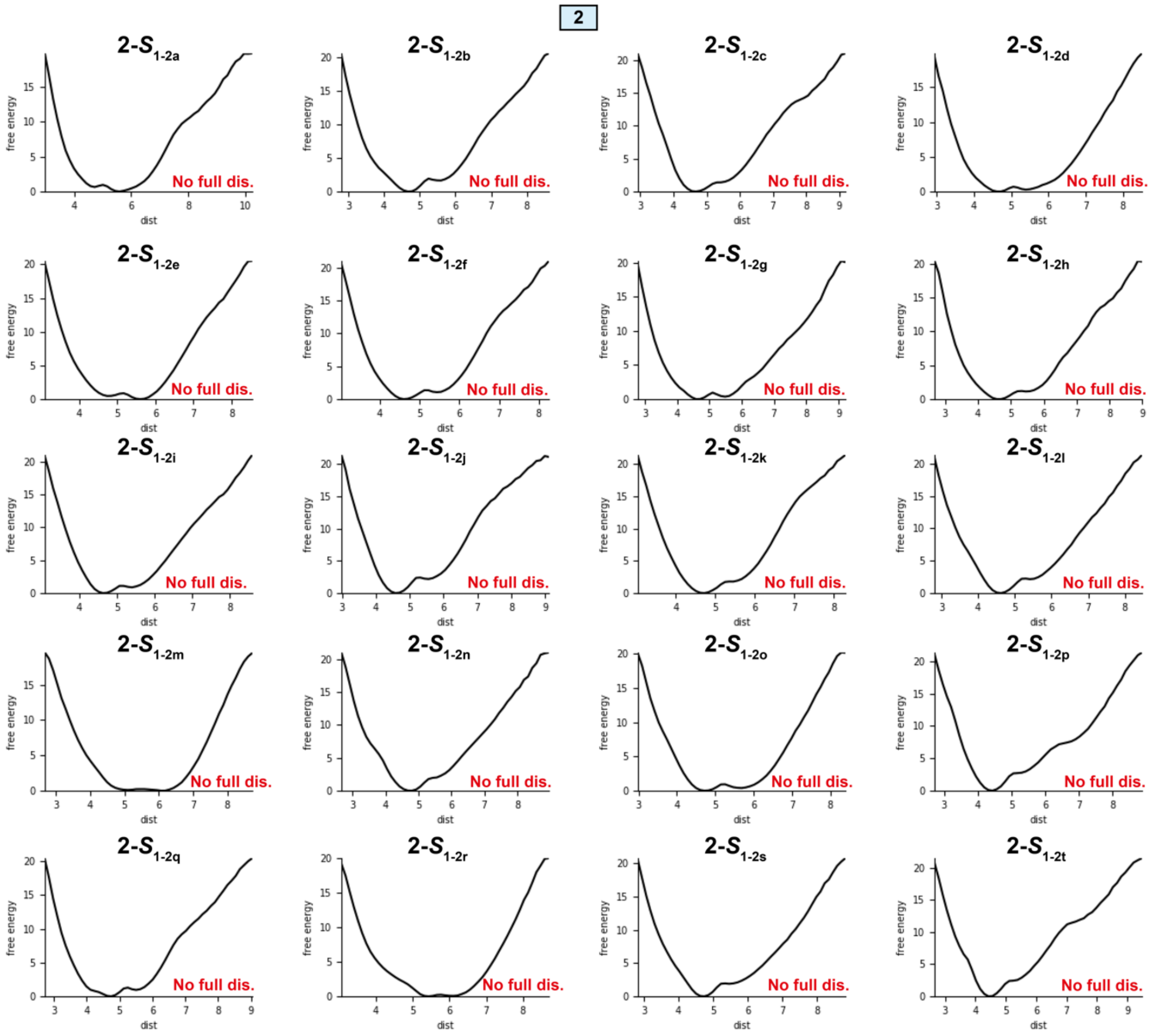

Supplementary Figure 23. **Well-tempered metadynamics simulations of 2-*S*$_1$ conformation II (20 replicates).** In the y-axis free energy and in the x-axis distance between the centre of mass of binding site residues and centre of mass of the inhibitor. Each simulation is 200 ns.

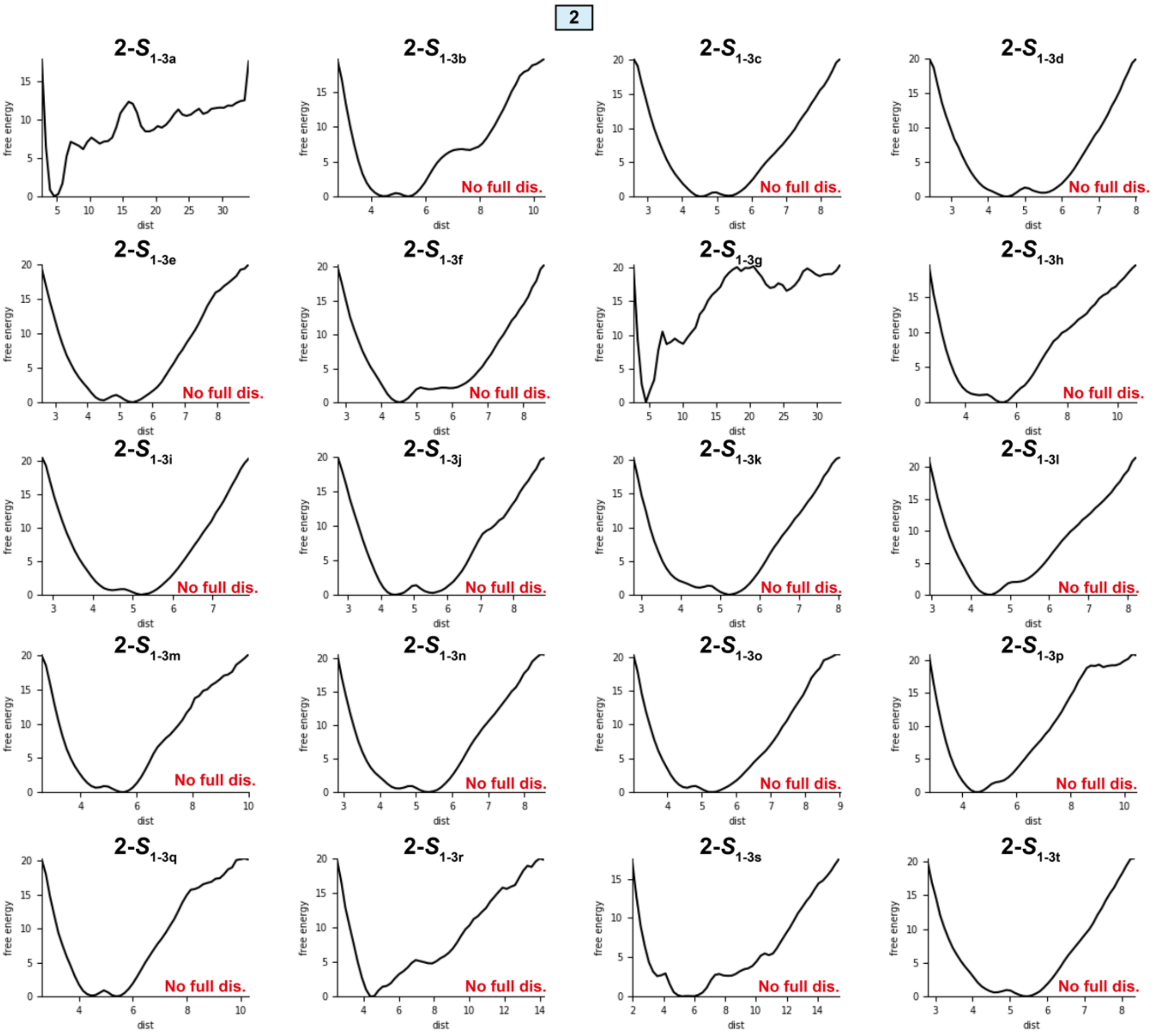

Supplementary Figure 24. **Well-tempered metadynamics simulations of 2-S_1 conformation III (20 replicates).** In the y-axis free energy and in the x-axis distance between the centre of mass of binding site residues and centre of mass of the inhibitor. Each simulation is 200 ns.

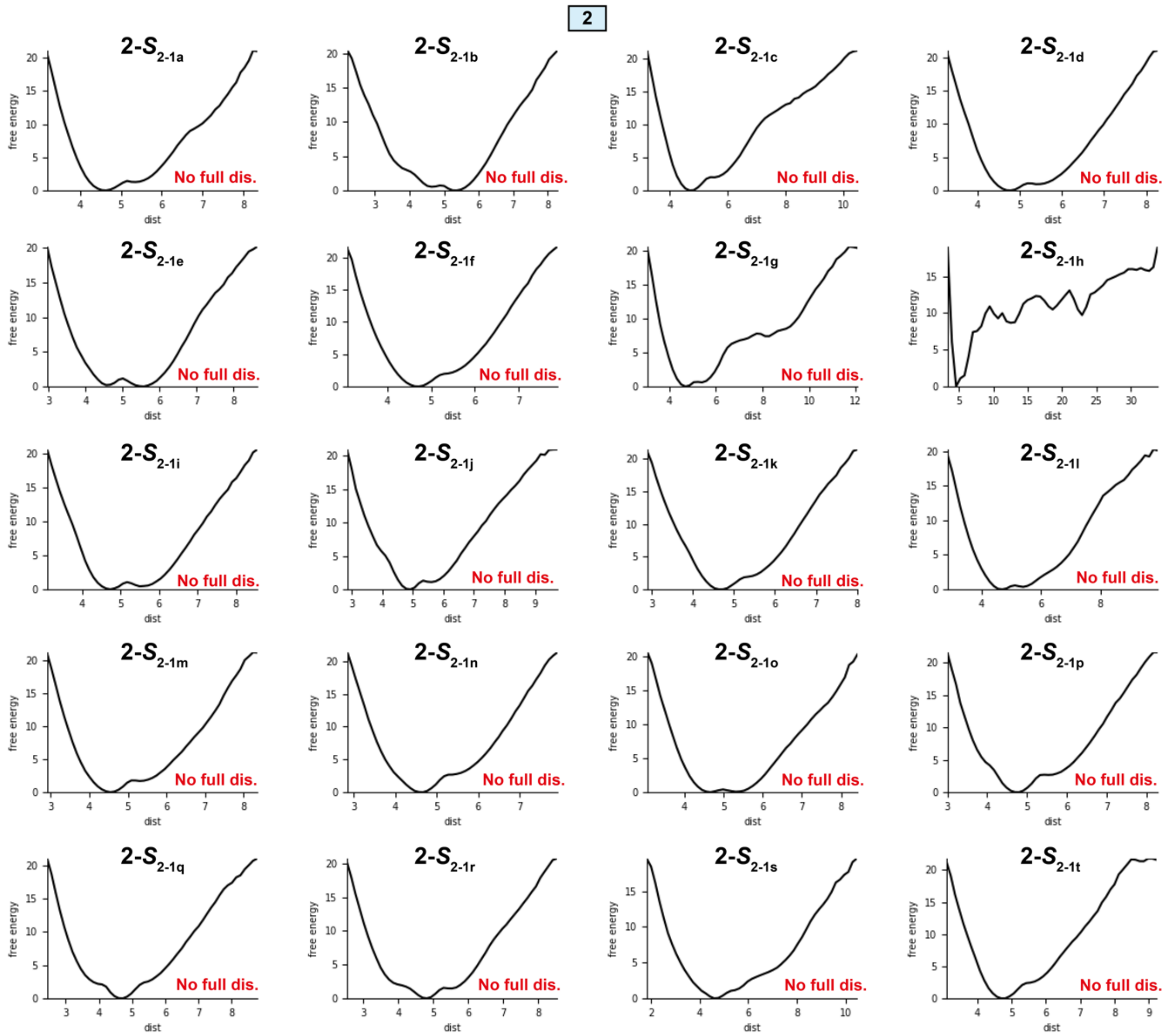

Supplementary Figure 25. **Well-tempered metadynamics simulations of 2-S_2 conformation I (20 replicates).** In the y-axis free energy and in the x-axis distance between the centre of mass of binding site residues and centre of mass of the inhibitor. Each simulation is 200 ns.

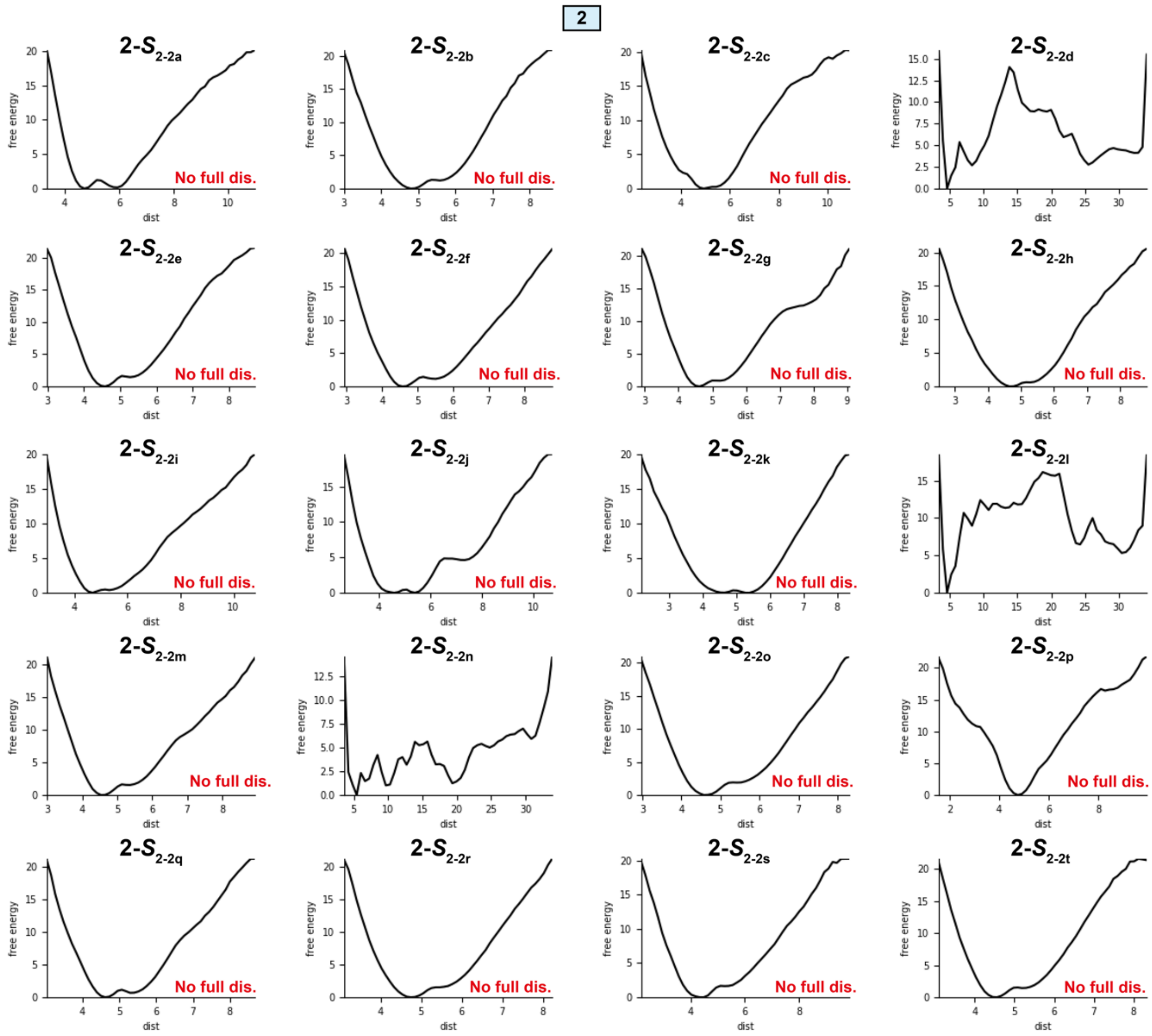

Supplementary Figure 26. **Well-tempered metadynamics simulations of 2-S_2 conformation II (20 replicates).** In the y-axis free energy and in the x-axis distance between the centre of mass of binding site residues and centre of mass of the inhibitor. Each simulation is 200 ns.

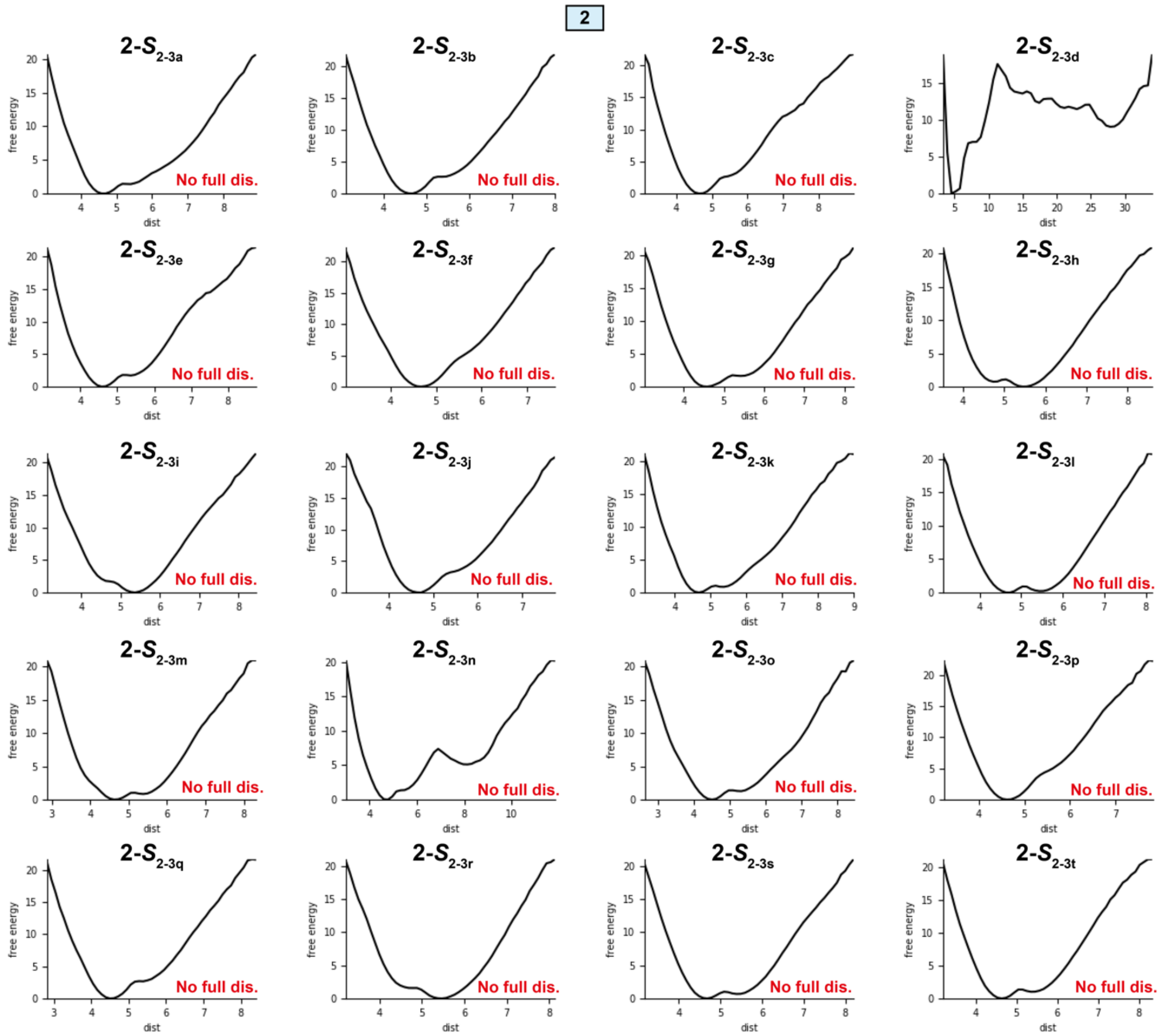

Supplementary Figure 27. **Well-tempered metadynamics simulations of 2-S_2 conformation III (20 replicates).** In the y-axis free energy and in the x-axis distance between the centre of mass of binding site residues and centre of mass of the inhibitor. Each simulation is 200 ns.

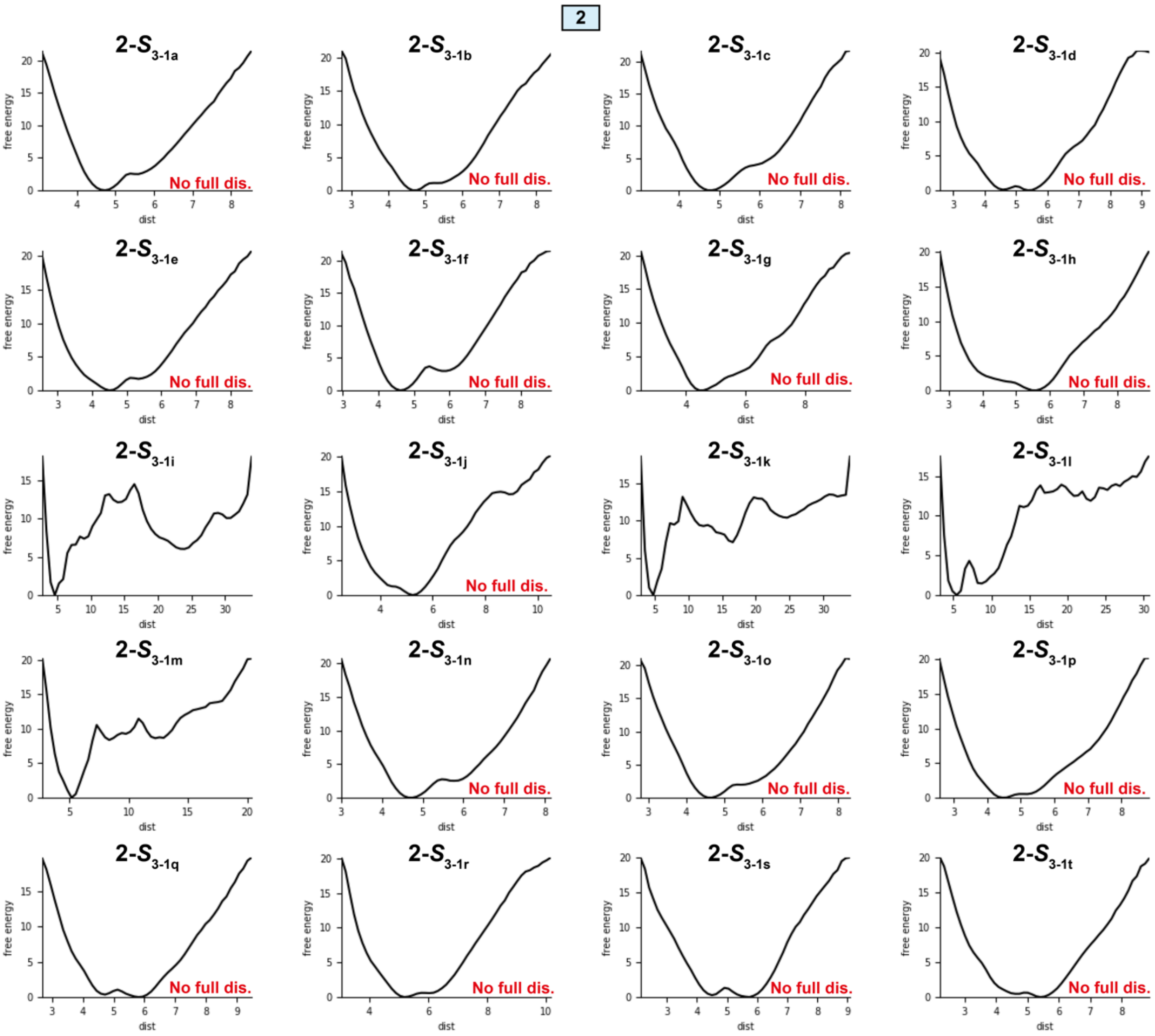

Supplementary Figure 28. **Well-tempered metadynamics simulations of 2-S_3 conformation I (20 replicates).** In the y-axis free energy and in the x-axis distance between the centre of mass of binding site residues and centre of mass of the inhibitor. Each simulation is 200 ns.

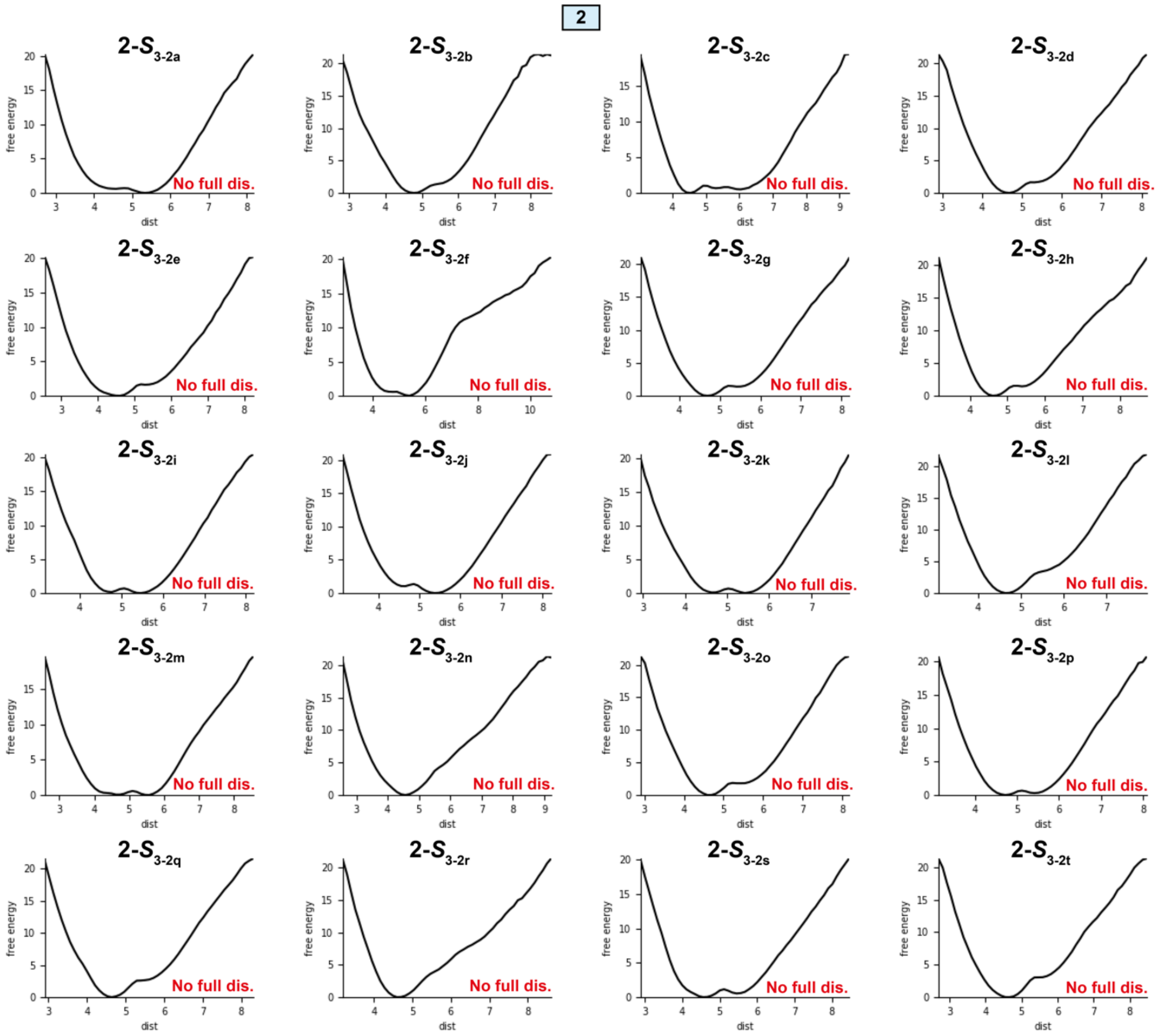

Supplementary Figure 29. **Well-tempered metadynamics simulations of 2-S_3 conformation II (20 replicates).** In the y-axis free energy and in the x-axis distance between the centre of mass of binding site residues and centre of mass of the inhibitor. Each simulation is 200 ns.

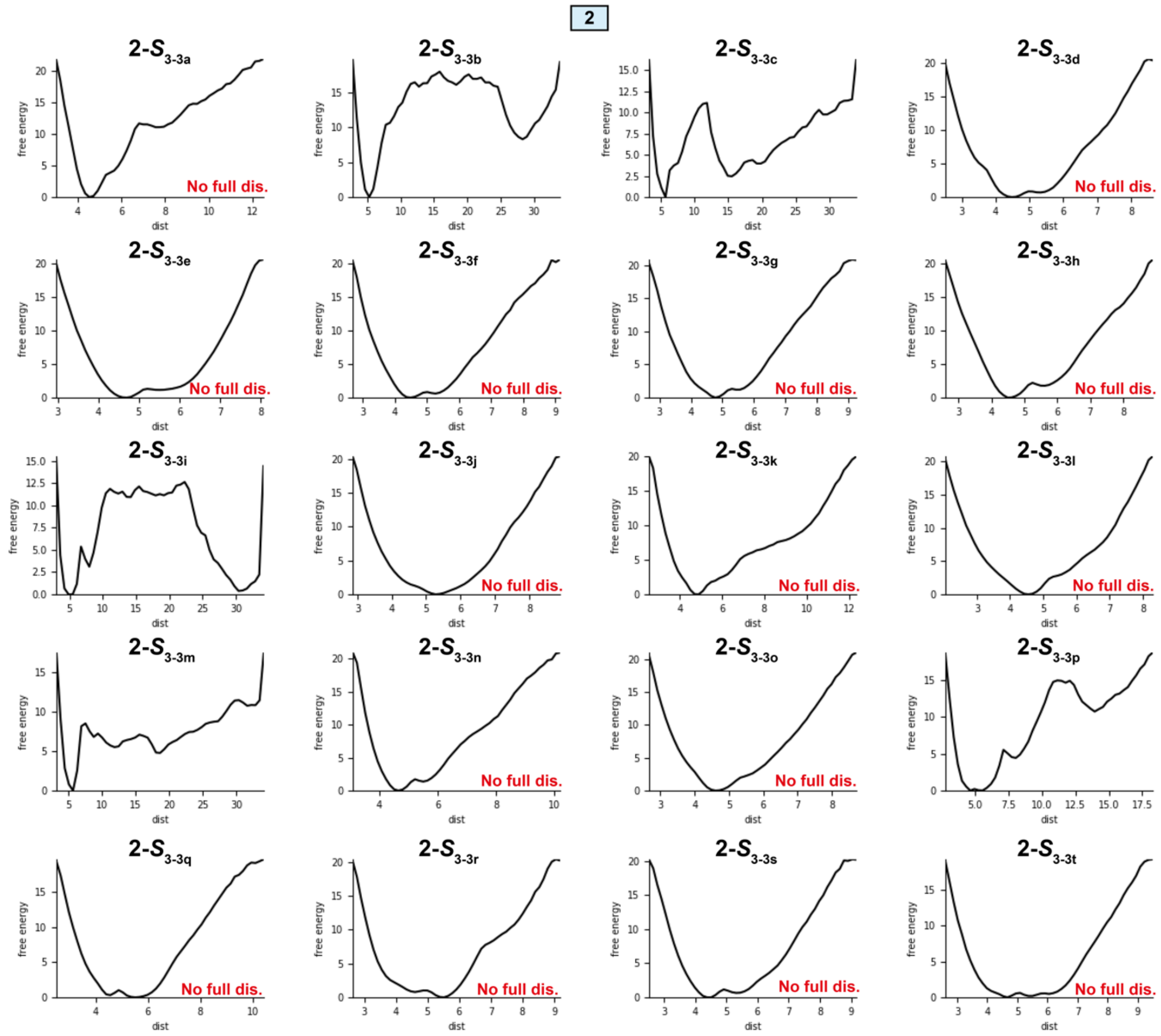

Supplementary Figure 30. **Well-tempered metadynamics simulations of 2-*S*$_3$ conformation III (20 replicates).** In the y-axis free energy and in the x-axis distance between the centre of mass of binding site residues and centre of mass of the inhibitor. Each simulation is 200 ns.

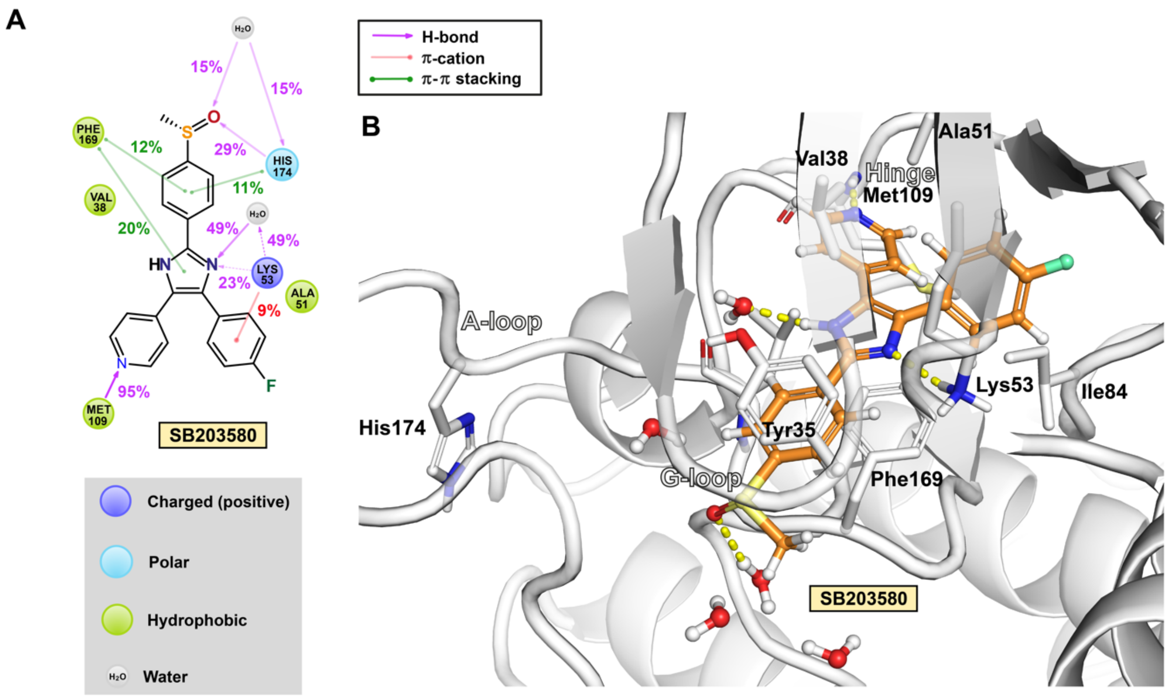

Supplementary Figure 31. **Key interactions observed in p38α MAPK–SB203580 complex simulations.** (**A**) 2D-representation of protein–ligand interaction frequencies. Displayed are the most frequent H-bond, π–cation and π–π stacking interaction frequencies (interactions with > 15% frequency are shown). In the simulations multiple varying interactions (mainly water mediated) are also observed with other residues such as Ser32, Ala34, Tyr35, Leu171, Arg178 Glu178 with varying frequencies among different replicas. (**B**) Binding mode of **SB203580** shown with the start conformation of the simulations. The key interaction residues and selected other residues are shown with sticks together with solvent molecules in the close proximity of the ligand. H-bonds shown with yellow dashed lines.

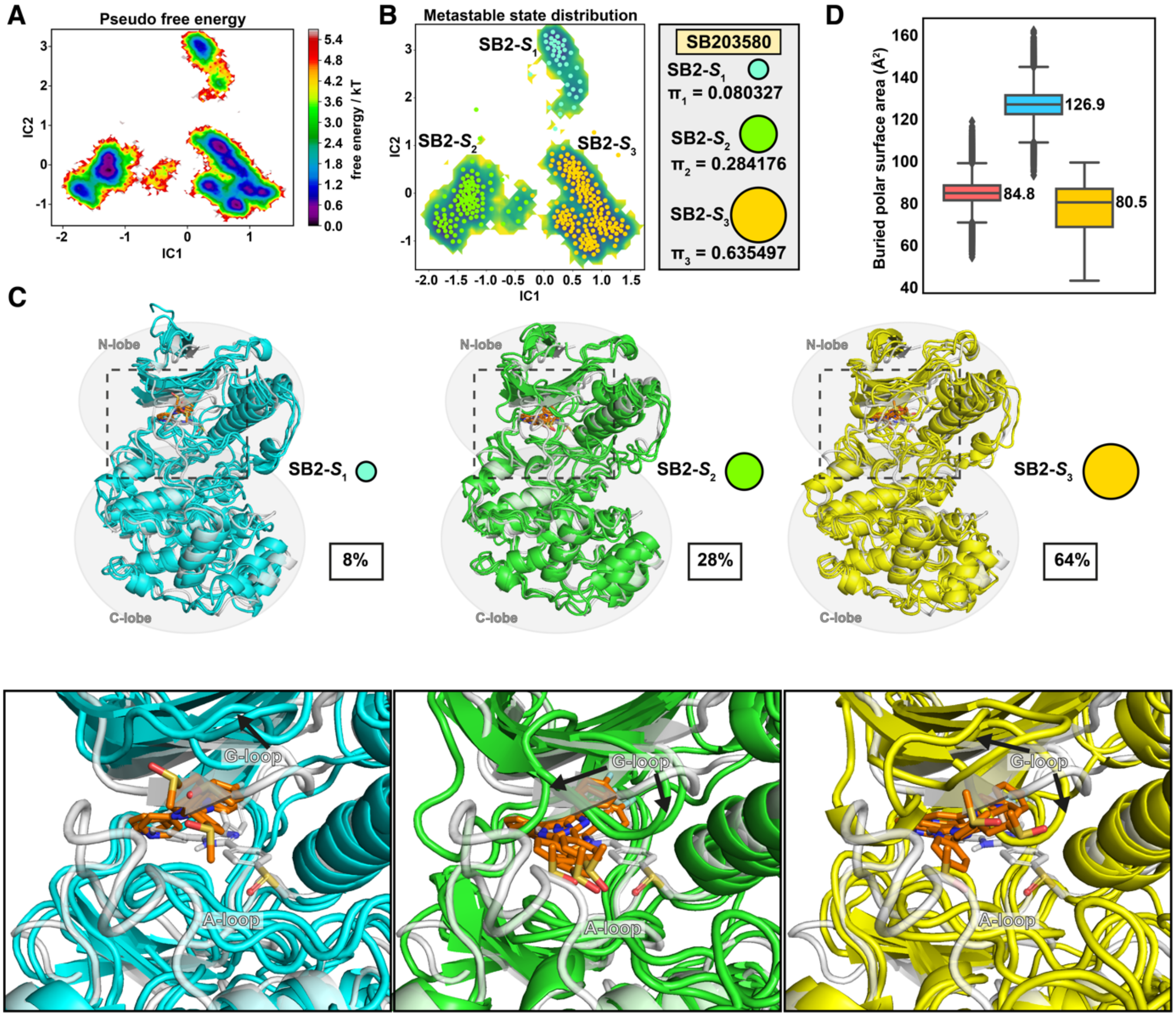

Supplementary Figure 32. **MSM metastable states of p38α MAPK–SB203580 complex.** (**A**) Pseudo free energy map of distributions along time-lagged independent components (ICs) 1 and 2. (**B**) Separation of the three metastable states (**SB2-S_1–SB2-S_3**) by PCCA++ analysis. (**C**) Representative conformations of the metastable states. Each metastable state (**SB2-S_i**) is illustrated with three representative structures (coloured cartoons) and the simulation starting conformation is shown as a reference in grey. Equilibrium probability (π_i) for each state is indicated together with circles, which with an area that is relative to state probability. In all states the ligand conformation is different than in the starting conformation. Within **SB2-S_1** and **SB2-S_3** the ligand conformation also varies among the conformations, while in **SB2-S_2** ligand appears to prefer less variation in its conformation. (**D**) Buried polar surface areas of 1, 2 and SB203580. Simulation data was analysed for each 1 ns i.e. data in **D** consist of 91,328, 86,505 and 91,090 individual data points for 1, 2 and SB203580, respectively. The black horizontal line in the box represents the median. Box displays the quartiles of the dataset (25%–75%) and whiskers the rest of the data with maximum 1.5 IQR. Outliers are indicated with black diamonds. Source data are provided as a Source Data file.

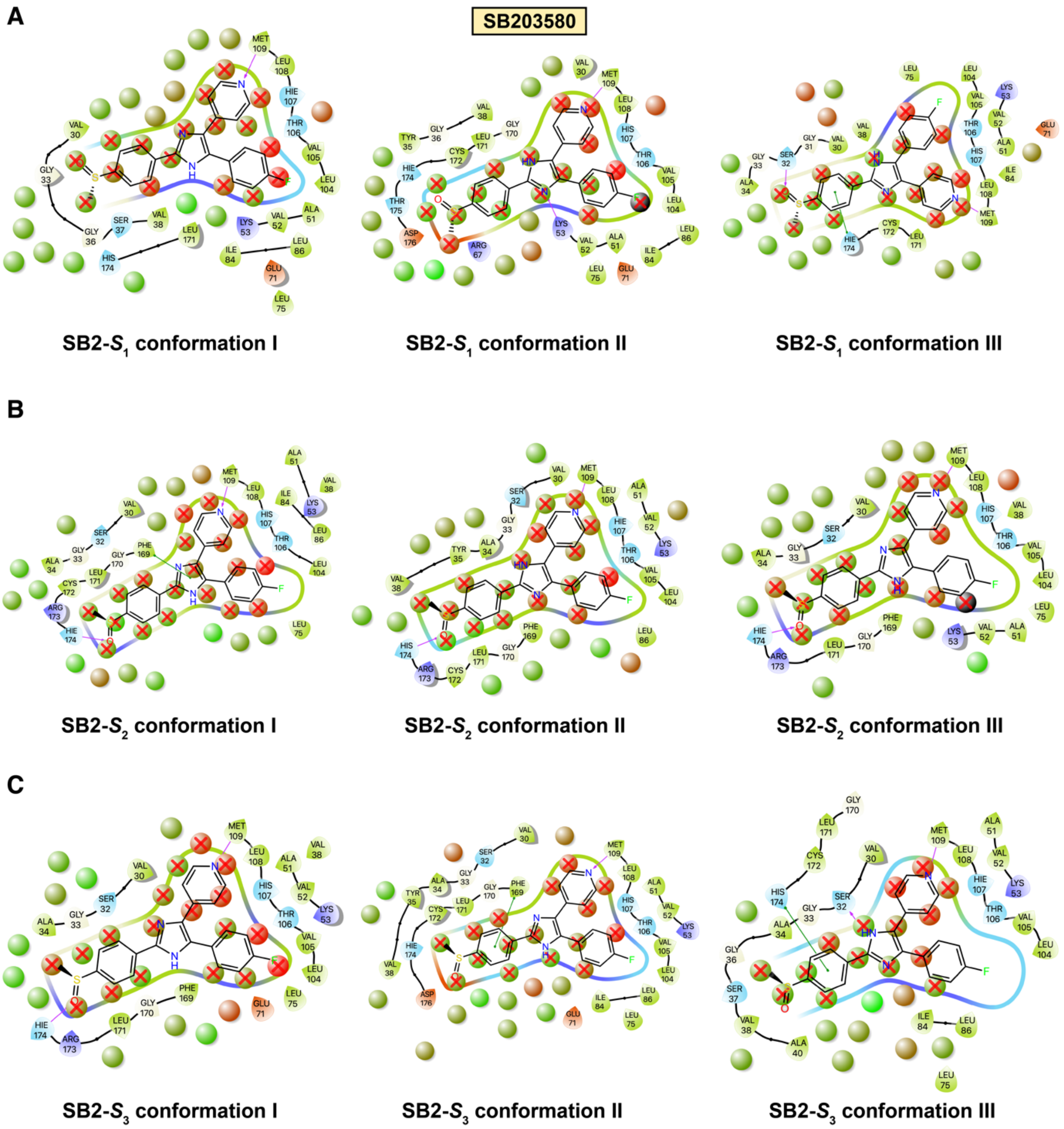

Supplementary Figure 33. **Hydration site displacement by SB203580.** 2D-represantation of the WaterMap results calculated for three conformations derived from (**A**) **SB2-S_1**, (**B**) **SB2-S_2** and (**C**) **SB2-S_3**. Hydration sites that would be displaced by **SB203580** are marked with red cross mark.

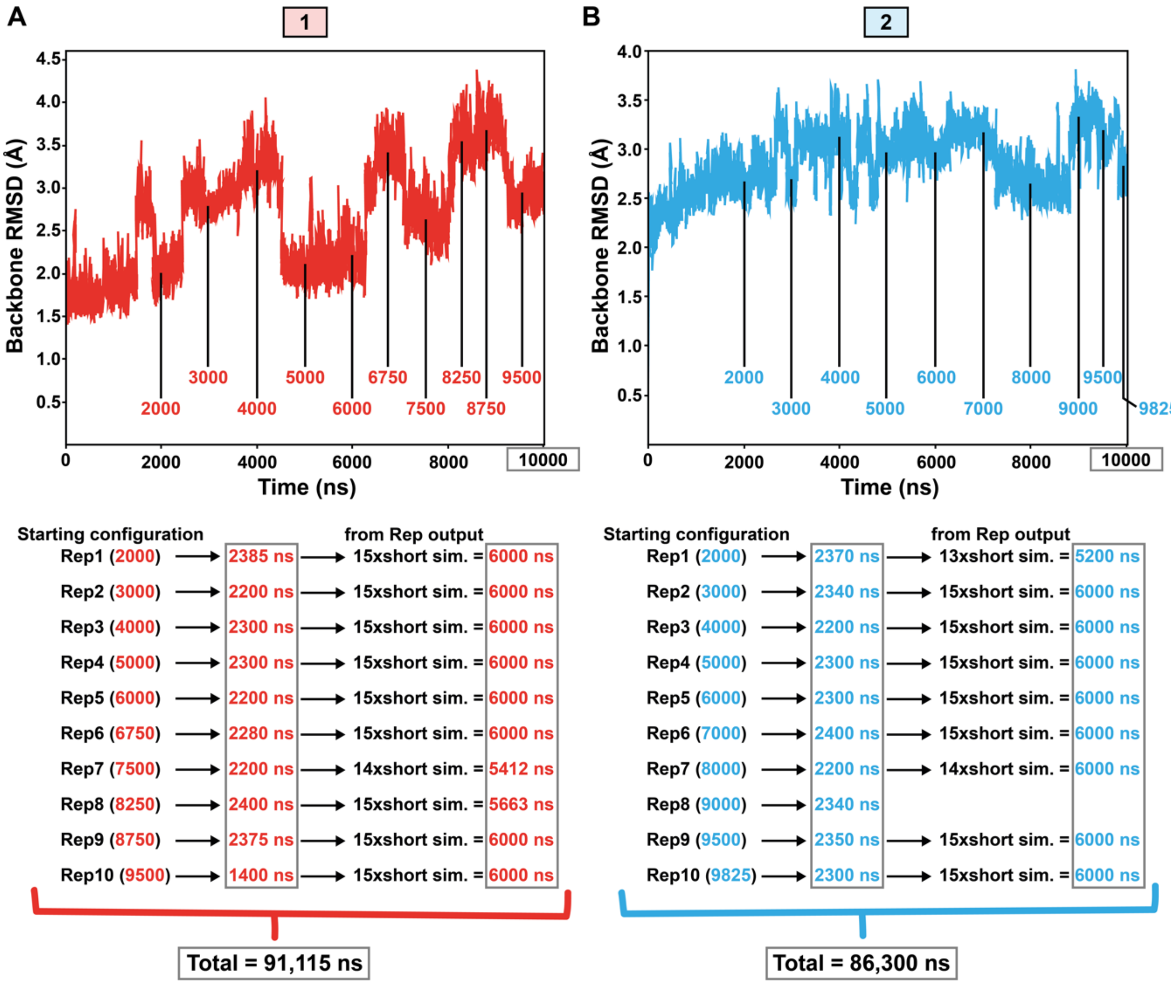

Supplementary Figure 34. **Outline of the conducted simulations (1/2).** First, a single 10 µs simulations were run for both **1** (**A**) and **2** (**B**). Starting from different protein configurations (based on the protein backbone RMSD), ten new replicas (Rep1–10) were generated. These new replicas were prepared with Protein Preparation Wizard and re-solvated (similarly as the initial simulations, see details in methods). From these replica simulation output conformations, further short simulations (with different seed numbers) were generated and conducted. Altogether, these simulations resulted in 91,115 ns for **1** and 86,300 ns for **2**.

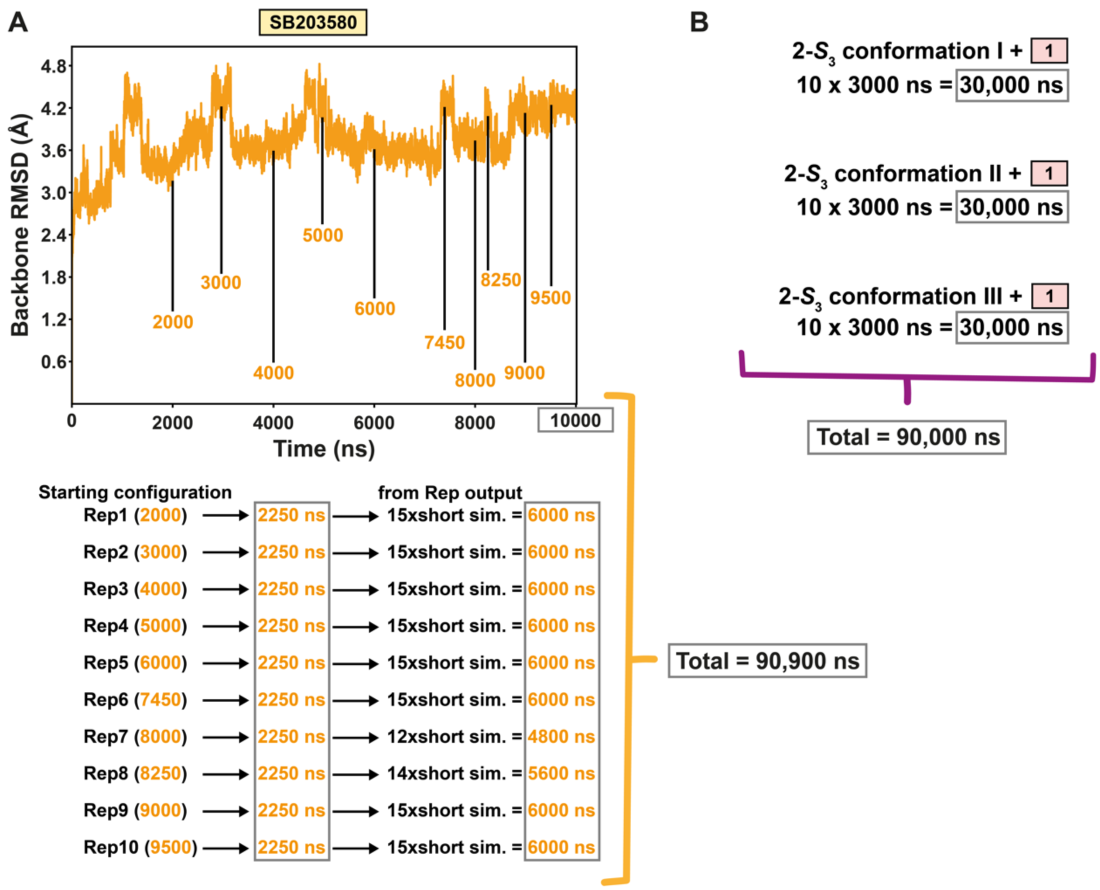

Supplementary Figure 35. **Outline of the conducted simulations (2/2).** First, a single 10 µs simulations was run for **SB203580** (**A**). Starting from different protein configurations (based on the protein backbone RMSD), ten new replicas (Rep1–10) were generated. These new replicas were prepared with Protein Preparation Wizard and re-solvated (similarly as the initial simulations, see details in methods). From these replica simulation output conformations, further short simulations (with different seed numbers) were generated and conducted. Altogether, these simulations resulted in 90,900 ns for **SB203580. (B)** Simulation of **1** in **2-S_3** conformations. Ten replicas for each of the three metastable state derived structures were conducted, resulting in a total of 90,000 ns.

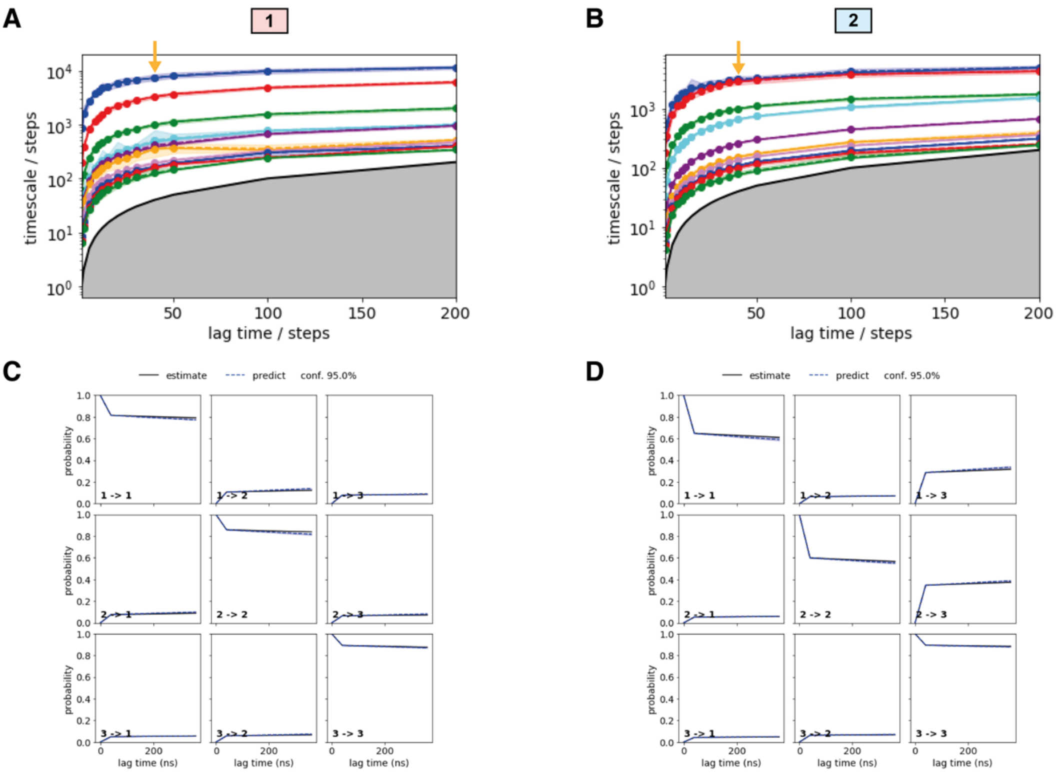

Supplementary Figure 36. **Validation of Markov State Models of 1 and 2.** In the selected lag times (40 ns, indicated with yellow arrows) the implied timescales are converged for **1** (**A**) and **2** (**B**). Chapman-Kolmogorov tests demonstrate that the models follow expected estimates for **1** (**C**) and **2** (**D**).

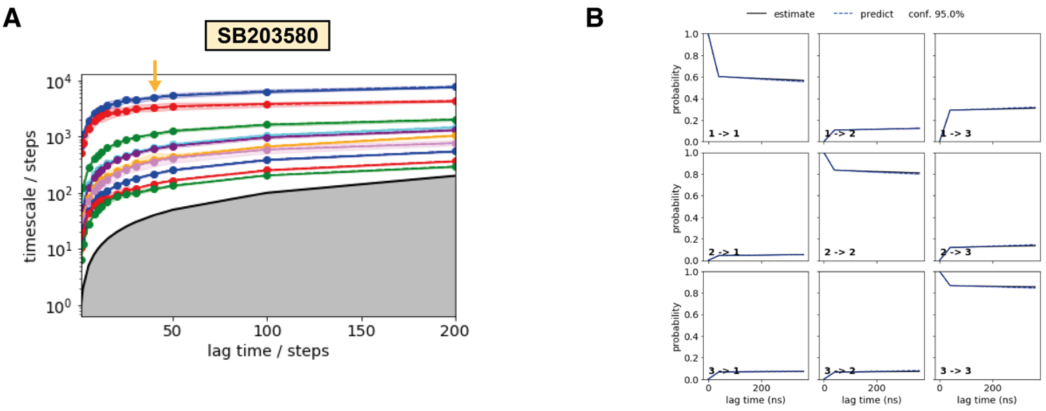

Supplementary Figure 37. **Validation of Markov State Model of SB203580.** In the selected lag times (40 ns, indicated with yellow arrow) the implied timescales are converged for **SB203580** (**A**). Chapman-Kolmogorov tests demonstrate that the model follow expected estimates for **SB230580** (**B**).

Supplementary Methods: Compound Synthesis

General Remarks and Instrumentation

All starting materials, reagents and solvents are commercially available and were used without further purification. Some intermediates were prepared using literature known procedures (cited where applicable). NMR spectra were recorded on a Bruker Avance 200 or with Bruker Avance 400 instruments. The spectra were measured in the indicated solvent and calibrated against the residual proton peak of the deuterated solvent. Chemical shifts (δ) are reported in parts per million. Mass spectra were obtained either from the Mass Spectrometry Department (FAB-MS), Institute of Organic Chemistry, Eberhard-Karls-Universitaet Tuebingen or from the Institute of Sciences, Department of Pharmaceutical Analytics and Bioanalytics. TLC-MS spectra were obtained from an Advion TLC-MS interface with electron spray ionization (ESI) in positive and/or negative mode. Instrument settings as follows: ESI voltage 3.50 kV, capillary voltage 187 V, source voltage 44 V, capillary temperature 250 °C, desolvation gas temperature 250 °C, gas flow 5 l/min nitrogen. GC-MS analyses were carried out on a Hewlett-Packard HP 6890 series GC system equipped with a HP-5MS capillary column (0.25 μm film thickness, 30 m × 250 μm) and a HP 5973 mass selective detector (EI ionization). Helium was used as carrier gas in the following temperature program: start at 160 °C and hold for 1 min, then increase to 240 °C and hold for 5 min, then increase to 270 °C and hold for 35 min. High resolution, high accuracy mass spectrometry (ESI-HRMS) was performed on a Bruker APEX II or a Sciex (Ontario, Canada) TripleTof 5600+ mass spectrometer with a Duospray source, coupled to a 1290 UHPLC from Agilent (Waldbron, Germany) equipped with an PAL-HTS autosampler from CTC (Zwingen, Switzerland). TLC analyses were performed on fluorescent silica gel 60 F254 plates (Merck) and visualized under UV at 254 and 366 nm. Column chromatography was performed on Davisil LC60A 20−45 μm silica from Grace Davison or Geduran Si60 63−200 μm silica from Merck using an Interchim PuriFlash 430 automated flash chromatography system. The purity of all final compounds was determined via HPLC and is >95% unless otherwise stated. The measurements were performed on a Hewlett-Packard HP 1090 series II LC or an Agilent 1100 series LC equipped with a UV diode array detector (DAD, detection at 230 and 254 nm). The chromatographic separation was performed on a Phenomenex Luna 5u C8 column (150 mm × 4.6 mm, 5 μm) at 25 °C oven temperature. The injection volume was 5 μL, and the gradient of the used method was the following (flow, 1.5 mL/min), with 0.01 M KH_2PO_4, pH 2.3 (solvent A), methanol (solvent B): from 40% B to 85% B in 8 min, 85% B for 5 min, from 85% to 40% B in 1 min, 40% B for 2 min, stop time 16 min.

The starting material **S01** for both compounds **1** and **2** was synthesized according to the previous described synthetic routes [1,2]. The compound **SB203580** is a widely known reference compound with broad commercial availability. The compound was synthesized according the already described procedures in patent literature (US5686455A)[3] and the analytical characterization of the final compound is in line with former reports and the commercial available material: ^{1}H NMR (400 MHz, DMSO) δ 13.13 (br s) and 13.04 (br s, 1H), 8.66 – 8.41 (m, 2H), 8.26 (d, J = 8.2 Hz, 2H), 7.81 (d, J = 8.4 Hz, 2H), 7.65 – 7.54 (m, 2H), 7.53 – 7.44 (m, 2H), 7.42 – 7.14 (m, 2H), 2.80 (s, 3H). Mass calcd. for $C_{21}H_{16}FN_3OS$: 377.10; found: (ESI+) m/z: 378.2 $[M+H]^+$ / (ESI-) m/z: 376.1 $[M-H]^-$, HPLC purity (254 nm): > 99.9 area%.

Synthesis of Compound 1

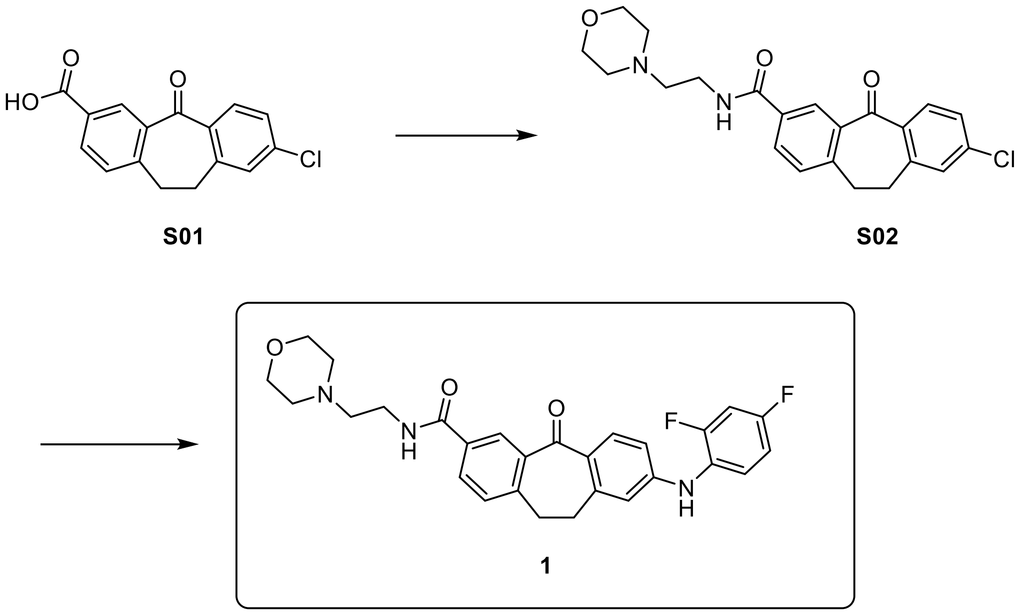

8-chloro-N-(2-morpholinoethyl)-5-oxo-10,11-dihydro-5H-dibenzo[a,d][7]annulene-3-carboxamide (**S02**). To a suspension of **S01**[1,2] (500 mg, 1.75 mmol) in THF (20 mL) was added CDI (502 mg, 3.10 mmol) at ambient temperature. After 1.5 hours was added 2-morpholinoethan-1-amine (676 mg, 5.20 mmol) and stirring was continued overnight. The mixture was then diluted with water and extracted with EtOAc (3 x 30 mL). The combined organic layers were washed with water, dried over Na_2SO_4 and evaporated to obtain the title substance as off-white to brown solid. Yield: 684 mg (98%). ^{1}H-NMR (200 MHz, $CDCl_3$) δ 8.35 (d, J = 2.1 Hz, 1H), 7.92-8.09 (m, 2H), 7.30-7.39 (m, 2H), 7.23-7.29 (m, 1H), 6.85 (s, 1H), 3.75 (t, J = 4.6 Hz, 4H), 3.51-3.64 (m, 2H), 3.09-3.36 (m, 4H), 2.62 (t, J = 5.9 Hz, 2H), 2.52 (t, J = 4.6 Hz, 4H). ^{13}C-NMR (50 MHz, $CDCl_3$) δ 192.9, 166.4, 145.1, 143.7, 138.9, 138.0, 133.4, 136.3, 132.7, 131.8, 130.1, 129.3, 128.7, 127.1, 66.9, 57.0, 53.4, 36.2, 34.7, 34.6. GC-MS EI m/z = 398 $[M]^+$

8-((2,4-difluorophenyl)amino)-N-(2-morpholinoethyl)-5-oxo-10,11-dihydro-5H-dibenzo[a,d][7]annulene-3-carboxamide (**1**). A screwtop reaction vial was charged with **S02** (620 mg, 1.6 mmol), 2,4-difluoroaniline (220 mg, 1.7 mmol), KO*t*Bu (630 mg, 5.6 mmol), $Pd(OAc)_2$ (50 mg, 0.22 mmol) and XPhos (140 mg, 0.29 mmol) under argon atmosphere. To this was added a mixture of toluene (10 mL) and *t*-BuOH (2 mL) and the reaction was heated to reflux for two hours. The reaction was poured on water and extracted with Et_2O (3 x 30 mL). The combined extracts were washed with brine, dried over Na_2SO_4 and evaporated to dryness. The residue was purified via flash chromatography (DCM / EtOH 95:5) and recrystallization (hexane / EtOAc) to afford the final compound as yellowish solid. Yield: 393 mg (50%). ^{1}H-NMR (200 MHz, DMSO) δ 8.58 (s, 1H), 8.51 (t , J = 5.7 Hz, 1H), 8.32 (d, J = 1.8 Hz, 1H), 7.99 (d, J = 8.7 Hz, 1H), 7.90 (dd, J = 7.8 Hz, 1.9 Hz, 1H), 7.29-7.48 (m, 3H), 7.09-7.16 (m, 1H), 6.75 (d, J = 8.7 Hz, 1H), 6.62 (s, 1H), 3.55 (t, J = 4.6 Hz, 4H), 3.25-3.45 (m, 2H), 2.94-3.19 (m, 4H), 2.31-2.46 (m, 6H). ^{13}C-NMR (100 MHz, DMSO) δ 190.4, 165.5, 158.5 (dd, J = 242.0 Hz, 11.6 Hz), 155.7 (dd, J =247.6 Hz, 12.8 Hz), 149.3, 145.2, 144.5, 138.9, 133.3, 132.8, 130.4, 129.1, 128.9, 127.1, 126.2 (dd, J = 9.4 Hz, 3.0 Hz), 124.8 (dd, J = 11.6 Hz, 3.0 Hz), 113.5, 111.8 (dd, J = 21.8 Hz, 3.6 Hz), 112.3, 104.9 (dd, J =26.6 Hz, 23.6 Hz), 66.1, 57.3, 53.2, 36.5, 35.4, 33.8. ESI-HRMS $[M+H]^+$ calculated: 492.20933, found: 492.20963.

Synthesis of Compound 2

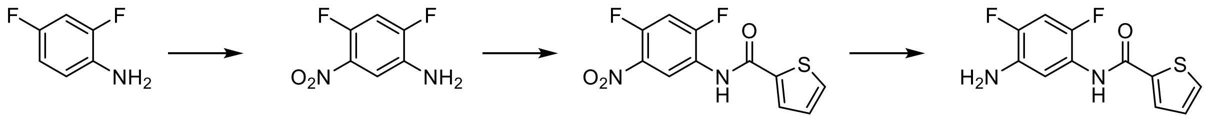

S03 S04 S05

2,4-Difluoro-5-nitroaniline (**S03**). To a cooled mixture of 2,4-difluoroaniline (2.0 g, 15.6 mmol) in concentrated sulfuric acid (20 mL) was added concentrated nitric acid (2 mL, ca. 2 eq.) dropwise in a rate that the internal temperature was kept below 5 °C. After complete addition, stirring was continued for 30 min before the reaction mixture was poured on Ice and the pH was set to pH 13 with NaOH under external cooling. The mixture was extracted with Et_2O (3 x 30 mL) and the combined extracts were dried over Na_2SO_4 and evaporated under reduced pressure. The title compound was obtained as brown solid and was used as isolated without further purification. Yield 2.41 g (89%). ^{1}H NMR (200 MHz, $CDCl_3$) δ 7.57 – 7.42 (m, 1H), 6.96 (t, *J* = 10.3 Hz, 1H), 3.88 (br s, 2H). ^{13}C NMR (50 MHz, $CDCl_3$) δ 153.7 (dd, *J* = 253.9, 10.0 Hz), 148.6 (dd, *J* = 258.8, 11.5 Hz), 134.5 – 133.1 (m), 131.9 (dd, *J* = 14.0, 3.2 Hz), 112.4 (dd, *J* = 6.5, 2.6 Hz), 106.3 (dd, *J* = 25.6, 24.6 Hz). FAB-MS m/z = 175.2 $[M+H]^+$

N-(2,4-difluoro-5-nitrophenyl)thiophene-2-carboxamide (**S04**). To a suspension of sodium hydride (60 wt% dispersion in min. oil, 69 mg, 1.72 mmol) in dry THF (10 mL) was added a solution of **S03** (300 mg, 1.72 mmol) in dry THF (5 mL) dropwise. After complete addition, stirring was continued for one hour at ambient temperature and was then cooled with an ice bath, before 2-thiophenecarbonyl chloride (277 mg, 1.89 mmol) was added. The cooling bath was removed and the reaction was stirred overnight at ambient temperature. The mixture was poured on water and extracted with EtOAc (3 x 20 mL) and the combined extracts were dried over Na_2SO_4 and evaporated. The residue was purified via flash chromatography (hexane / EtOAc 80:20) to obtain the title compound as brownish solid. Yield: 320 mg (65%). ^{1}H NMR (200 MHz, DMSO) δ 10.46 (s, 1H), 8.54 – 8.42 (m, 1H), 8.08 – 8.00 (m, 1H), 7.94 – 7.79 (m, 2H), 7.33 – 7.21 (m, 1H). TLC-MS ESI m/z = 282.9 $[M-H]^-$

N-(5-amino-2,4-difluorophenyl)thiophene-2-carboxamide (**S05**). To a refluxing solution of **S04** (290 mg, 1.02 mmol) in EtOH (14 mL) was added $SnCl_2$ dihydrate (1151 mg, 5.1 mmol) portionwise over 4 hours. TLC indicated complete conversion after 5 hour and an excess of solid $NaHCO_3$ was added carefully (foaming !) to the warm solution. The alcohol was removed on the rotavap and the solid residue was resuspended in EtOAc (40 mL) followed by stirring at ambinet temperature for 15 min. The suspension was filtered over a bed of celite and the filtrate was evaporated under reduced pressure. The crude product was purified via flash chromatography (hexane / EtOAc 50:50) to afford the title substance as off-white solid. Yield: 240 mg (93%). ^{1}H NMR (200 MHz, DMSO-d6) δ 9.92 (s, 1H), 8.02 – 7.78 (m, 2H), 7.27 – 7.03 (m, 2H), 7.00 – 6.85 (m, 1H), 5.07 (s, 2H). TLC-MS ESI m/z = 252.9 $[M-H]^-$

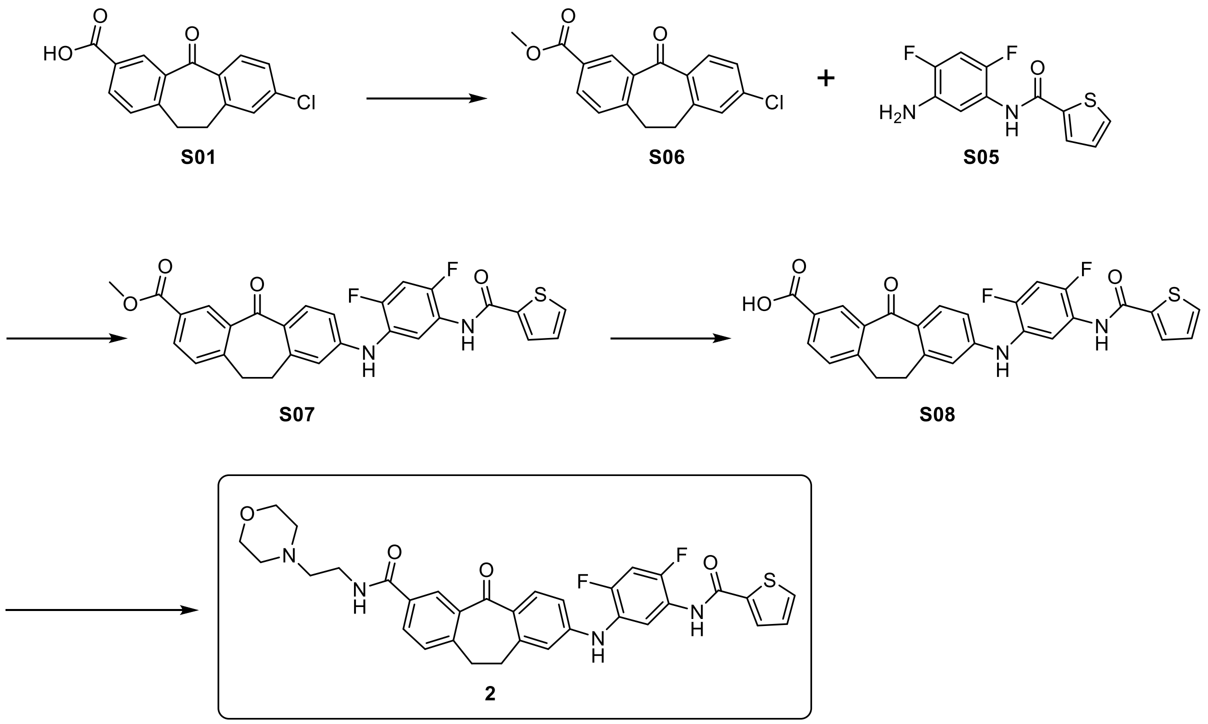

methyl 8-chloro-5-oxo-10,11-dihydro-5H-dibenzo[a,d][7]annulene-3-carboxylate (**S06**). To a solution of **S01**[1,2] (600 mg, 2.09 mmol) was added a catalytical amount of concentrated hydrochloric acid (0.5 mL) and the mixture was heated to reflux until TLC indicated complete conversion. The majority of the alcohol was removed under reduced pressure and the residue was taken up in EtOAc (30 mL). The organic phase was washed with 1 N aqueous NaOH and brine prior to drying over Na_2SO_4 and evaporation. The product was obtained as off-white solid. Yield: 620 mg (99%). ^1H NMR (400 MHz, $CDCl_3$) δ 8.65 (d, *J* = 1.9 Hz, 1H), 8.09 (dd, *J* = 7.9, 1.9 Hz, 1H), 8.00 (d, *J* = 8.5 Hz, 1H), 7.34 – 7.28 (m, 2H), 7.24 (d, *J* = 2.0 Hz, 1H), 3.93 (s, 3H), 3.27 – 3.13 (m, 4H). ^{13}C NMR (101 MHz, $CDCl_3$) δ 193.2, 166.4, 146.5, 143.7, 138.9, 138.6, 136.4, 133.3, 132.8, 132.4, 129.8, 129.4, 129.2, 127.3, 52.4, 34.8, 34.7. TLC-MS ESI m/z = 301.3 $[M+H]^+$

methyl 8-((2,4-difluoro-5-(thiophene-2-carboxamido)phenyl)amino)-5-oxo-10,11-dihydro-5H-dibenzo [a,d][7]annulene-3-carboxylate (**S07**). A screwtop reaction vial was charged with **S06** (99 mg, 0.33 mmol), **S05** (92 mg, 0.36 mmol), Cs_2CO_3 (161 mg, 0.50 mmol), $Pd(OAc)_2$ (7 mg, 0.03 mmol) and XPhos (47 mg, 0.1 mmol) under argon atmosphere. A degassed mixture (5:1) dioxane and *t*-BuOH (4.5 mL) was added and the reaction was heated to 60°C until TLC indicated complete conversion. After cooling to ambient temperature, the mixture was filtered over celite and washed with DCM, EtOAc and MeOH. The filtrate was evaporated and the residue was purified via flash chromatography (hexane / EtOAc) to obtain the title substance as yellow solid. Yield: 120 mg (70%). ^1H-NMR (200 MHz, $CDCl_3$) δ 8.64 (d, J = 1.8 Hz, 1H), 8.52 – 8.37 (m, 1H), 8.18 (d, J = 8.7 Hz, 1H), 8.05 (dd, J = 7.7, 1.8 Hz, 1H), 7.89 – 7.80 (m, 1H), 7.70 – 7.54 (m, 2H), 7.30 (s, 1H), 7.20 – 7.07 (m, 1H), 7.07 – 6.86 (m, 2H), 6.83 – 6.74 (m, 1H), 6.00 (s, 1H), 3.92 (s, 3H), 3.32 – 3.11 (m, 4H). ^{13}C-NMR (50 MHz, $CDCl_3$) δ 207.2, 191.7, 166.7, 160.0, 150.7 (dd, J = 244.5, 9.3 Hz), 148.5 (dd, J = 244.3, 12.2 Hz), 145.3, 139.6, 138.5, 134.1, 132.6, 132.3, 131.6, 129.3, 129.1 (d, J = 2.4 Hz), 128.8, 128.1, 125.1 (dd, J = 12.2, 3.5 Hz), 122.37 (dd, J = 11.4, 3.8 Hz); 120.1, 117.9, 116.2, 115.1, 113.7, 104.3 (t, J = 24.7 Hz), 52.2, 35.8, 34.9. ESI-HRMS $[M+H]^+$ calculated: 519.1185, found: 519.1189.

8-((2,4-difluoro-5-(thiophene-2-carboxamido)phenyl)amino)-5-oxo-10,11-dihydro-5H-dibenzo[a,d][7]annulene-3-carboxylic acid (**S08**). To a solution of **S07** (115 mg, 0.22 mmol) in MeOH

(4.5 mL) was added 1N aqueous KOH (0.5 mL) and the mixture was heated to reflux until TLC indicated complete conversion. The volatiles were removed under reduced pressure, the residue was taken up in water, acidified with diluted HCl and extracted with EtOAc (3 x 20 mL). The combined extracts were washed with brine, dried over Na_2SO_4 and evaporated to dryness. The residue was purified via flash chromatography (DCM / MeOH + formic acid) to obtain the title compound as yellow solid. Yield: 110 mg (98%). ^1H NMR (400 MHz, Acetone) δ 9.30 (s, 1H), 8.64 (s, 1H), 8.15 – 8.05 (m, 3H), 7.99 – 7.94 (m, 1H), 7.85 – 7.76 (m, 2H), 7.45 (d, J = 7.9 Hz, 1H), 7.29 – 7.17 (m, 2H), 7.02 – 6.95 (m, 1H), 6.90 (s, 1H), 3.28 – 3.13 (m, 4H). ^{13}C-NMR (100 MHz, Acetone) δ 191.2, 167.2, 161.1, 149.8, 147.8, 146.2, 140.5, 140.1, 134.6, 133.3, 133.0, 132.6, 130.2, 130.0 (d, J = 3.3 S33 Hz), 129.4, 128.8, 125.8 (dd, J = 13.0, 3.9 Hz), 123.3 (dd, J = 13.2, 3.3 Hz), 121.2, 121.1, 115.4, 114.0, 105.4 (t, J = 25.0 Hz), 36.5, 35.4. TLC-MS ESI m/z = 503.0 $[M-H]^-$

N-(2,4-difluoro-5-((7-((2-morpholinoethyl)carbamoyl)-5-oxo-10,11-dihydro-5H-dibenzo[a,d][7]annulen-2-yl)amino)phenyl)thiophene-2-carboxamide (**2**). To a solution of S08 (28 mg, 0.06 mmol) in dry DMF (3 mL) was added CDI (19 mg, 0.12 mmol) under argon atmosphere and the reaction was heated to 50 °C for one hour. Subsequently was added 2-morpholinoethan-1-amine (19 mg, 0.15 mmol) and stirring was continued overnight. The mixture was then poured on water and extracted with EtOAc (3 x 20 mL). The combined extracts were washed with brine, dried over Na2SO4 and evaporated. The residue was purified via flash chromatography (DCM / MeOH+NH_3 (95:5)) to obtain the final compound as yellow solid. Yield: 30 mg (85%). ^1HNMR (400 MHz, $CDCl_3$) δ 8.46 (t, J = 8.4 Hz, 1H), 8.30 (d, J = 1.4 Hz, 1H), 8.18 (d, J = 8.7 Hz, 1H), 8.01 – 7.92 (m, 1H), 7.88 (s, 1H), 7.62 (dd, J = 28.8, 4.1 Hz, 2H), 7.30 (d, J = 7.9 Hz, 1H), 7.17 – 7.12 (m, 1H), 7.05 – 6.96 (m, 1H), 6.96 – 6.87 (m, 2H), 6.81 (s, 1H), 6.06 (s, 1H), 3.86 – 3.69 (m, 4H), 3.63 – 3.52 (m, 2H), 3.22 – 3.08 (m, 4H), 2.63 – 2.59 (m, 2H), 2.51 (s, 4H). ^{13}C-NMR (100 MHz, $CDCl_3$) δ 191.7, 166.9, 159.9, 150.6 (dd, J = 248.3, 8.5 Hz), 147.4, 147.0 (d, J = 11.1 Hz), 145.5, 145.3, 139.2, 138.6, 134.3, 133.2, 131.6, 131.4, 129.5, 129.1, 128.6, 128.1, 125.3 (dd, J = 11.7, 3.1 Hz), 122.7 (dd, J = 11.1, 3.7 Hz), 115.7, 115.3, 114.0, 104.2 (d, J = 24.5 Hz), 104.1, 77.2, 67.1, 57.2, 53.6, 36.4, 36.0, 34.8. ESI-HRMS $[M-H]^+$ calculated: 617.2029, found: 617.2018.

Supplementary References

1. Fischer, S. *et al.* Dibenzosuberones as p38 mitogen-activated protein kinase inhibitors with low ATP competitiveness and outstanding whole blood activity. *J Med Chem* 56, 241–253 (2013).

2. Walter, N. M. *et al.* Design, Synthesis, and Biological Evaluation of Novel Type I1/2 p38*α* MAP Kinase Inhibitors with Excellent Selectivity, High Potency, and Prolonged Target Residence Time by Interfering with the R-Spine. *J. Med. Chem.* 60, 8027–8054 (2017).

3. Adams, J. L., Gallagher, T. F., Lee, J. C. & White, J. R. Imidazole derivatives and their use as cytokine inhibitors. US Patent: US5686455A (1997).
