# Supplementary material for: Decisive role of water and protein dynamics in residence time of p38α MAP kinase inhibitors
Source: Nat Commun. 2022 Jan 28;13:569. doi: 10.1038/s41467-022-28164-4 (PMC8799644; doi:10.1038/s41467-022-28164-4)
Supplement: Supplementary file 3 — Reporting Summary [file 41467_2022_28164_MOESM3_ESM.pdf]

nature portfolio

Corresponding author(s): *Double-blind peer review submissions: write DBPR and your manuscript number here instead of author names.*

Last updated by author(s): *YYYY-MM-DD*

Reporting Summary

Nature Portfolio wishes to improve the reproducibility of the work that we publish. This form provides structure for consistency and transparency in reporting. For further information on Nature Portfolio policies, see our Editorial Policies and the Editorial Policy Checklist.

Statistics

For all statistical analyses, confirm that the following items are present in the figure legend, table legend, main text, or Methods section.

n/a	Confirmed	
☐	☒	The exact sample size (*n*) for each experimental group/condition, given as a discrete number and unit of measurement
☐	☒	A statement on whether measurements were taken from distinct samples or whether the same sample was measured repeatedly
☐	☒	The statistical test(s) used AND whether they are one- or two-sided<br>*Only common tests should be described solely by name; describe more complex techniques in the Methods section.*
☒	☐	A description of all covariates tested
☐	☒	A description of any assumptions or corrections, such as tests of normality and adjustment for multiple comparisons
☐	☒	A full description of the statistical parameters including central tendency (e.g. means) or other basic estimates (e.g. regression coefficient) AND variation (e.g. standard deviation) or associated estimates of uncertainty (e.g. confidence intervals)
☐	☒	For null hypothesis testing, the test statistic (e.g. *F*, *t*, *r*) with confidence intervals, effect sizes, degrees of freedom and *P* value noted<br>*Give P values as exact values whenever suitable.*
☒	☐	For Bayesian analysis, information on the choice of priors and Markov chain Monte Carlo settings
☒	☐	For hierarchical and complex designs, identification of the appropriate level for tests and full reporting of outcomes
☒	☐	Estimates of effect sizes (e.g. Cohen's *d*, Pearson's *r*), indicating how they were calculated

Our web collection on statistics for biologists contains articles on many of the points above.

Software and code

Policy information about availability of computer code

Data collection	Maestro (versions 2018-4 and 2020-2) (Schrödinger, LLC, New York, NY, 2020)
Data analysis	Maestro version 2020-2 (Schrödinger, LLC, New York, NY, 2020); trajectory_asl_monitor.py script (Schrödinger LLC); event_analysis.py (Schrödinger LLC); analyze_simulation.py (Schrödinger LLC); Simulation Interaction Analysis tool (Schrödinger LLC);<br>PyEMMA 2.5.7; Jupyter notebooks used for MSM generation with PyEMMA2 are freely available at https://doi.org/10.5281/zenodo.5770578<br>Graphpad Prism version 7.0.0 for Windows (GraphPad Software, San Diego, CA, USA)<br>MetaXpress® Custom Module Editor Software (64 bit, 6.2.3.733, Molecular Devices)

For manuscripts utilizing custom algorithms or software that are central to the research but not yet described in published literature, software must be made available to editors and reviewers. We strongly encourage code deposition in a community repository (e.g. GitHub). See the Nature Portfolio guidelines for submitting code & software for further information.

Data

Policy information about availability of data

All manuscripts must include a data availability statement. This statement should provide the following information, where applicable:

- Accession codes, unique identifiers, or web links for publicly available datasets
- A description of any restrictions on data availability
- For clinical datasets or third party data, please ensure that the statement adheres to our policy

The original Desmond raw-trajectories, PDB-coordinates for the energy minimized metastable state derived structures and raw-trajectories of the well-tempered metadynamics simulations generated and analysed during the current study have been deposited in the Zenodo repository and are freely available at: https://doi.org/10.5281/zenodo.4568113 (compound 1); https://doi.org/10.5281/zenodo.4572444 (compound 1); https://doi.org/10.5281/zenodo.4561797 (compound

2); https://doi.org/10.5281/zenodo.4563896 (compound 2); https://doi.org/10.5281/zenodo.5563359 (SB203580); https://doi.org/10.5281/zenodo.5563655 (SB203580); https://doi.org/10.5281/zenodo.5564118 (compound 1 simulated in compound 2 metastable state 2-S3); https://doi.org/10.5281/zenodo.5564208 (compound 1 simulated in compound 2 metastable state 2-S3; dataset: VIII); https://doi.org/10.5281/zenodo.5564586 (well-tempered metadynamics simulations of compounds 1 and 2); https://doi.org/10.5281/zenodo.5570882 (well-tempered metadynamics simulations of compounds 1 and 2) https://doi.org/10.5281/zenodo.5571352 (well-tempered metadynamics simulations of compounds 1 and 2). Other data generated in this study are provided in the Supplementary Information and Source data file.
3que [http://doi.org/10.2210/pdb3QUE/pdb]
5tbe [http://doi.org/10.2210/pdb5TBE/pdb]
6zqs [http://doi.org/10.2210/pdb6ZQS/pdb]

Field-specific reporting

Please select the one below that is the best fit for your research. If you are not sure, read the appropriate sections before making your selection.

- [x] Life sciences
- [ ] Behavioural & social sciences
- [ ] Ecological, evolutionary & environmental sciences

For a reference copy of the document with all sections, see nature.com/documents/nr-reporting-summary-flat.pdf

Life sciences study design

All studies must disclose on these points even when the disclosure is negative.

Sample size	Sample sizes of 300 - 1000 cell per analyzed measurements were chosen as those are suffcient for statistical analysis. Any details on statistics are given in the method section.
Data exclusions	As stated, cells with ratios within the 10 - 90 percentiles of all measured cells were taken into account for statistical analysis.
Replication	All cellular experiments were performed in 3 individual replicates and 300 - 1000 cells were analyzed for each condition. All replications were successful.<br>MD simulations were run in 1–15 replicates for each individual starting configurations (see details in Supplementary Figures 34 and 35), a few short simulations failed during the run due to a technical error at the supercomputer (these were not repeated); well-tempered metadynamics simulations were conducted with 20 independent replicates for each MSM derived structures, with all replications being successful.
Randomization	Since we used cells stably expressing MK2-GFP, all cells within the 10 - 90 percentiles were taken into account. Thus, randomization was not applicable.
Blinding	Investigators were not blinded. Cells were subjected to automated quantitative measurements of the distribution of the fluorescent signal, therefore results were not affected by subjective bias.

Reporting for specific materials, systems and methods

We require information from authors about some types of materials, experimental systems and methods used in many studies. Here, indicate whether each material, system or method listed is relevant to your study. If you are not sure if a list item applies to your research, read the appropriate section before selecting a response.

Materials & experimental systems

n/a	Involved in the study	
x		Antibodies
	x	Eukaryotic cell lines
x		Palaeontology and archaeology
x		Animals and other organisms
x		Human research participants
x		Clinical data
x		Dual use research of concern

Methods

n/a	Involved in the study	
x		ChIP-seq
x		Flow cytometry
x		MRI-based neuroimaging

Eukaryotic cell lines

Policy information about cell lines

Cell line source(s)	DLD1 cells were obtained from ATCC (CCL-221), HCT116 cells were obtained from ATCC (CCL-247)
Authentication	Since this study does not include cell line specific analysis, all cell lines were used without additional authentication.
Mycoplasma contamination	Both cell lines were tested negative for mycoplasma using the PCR mycoplasma kit Venor GeM Classic (Minerva Biolabs, cat # 11-1025) and the Taq DNA Polymerase (Minerva Biolabs, cat # 53-0100)
Commonly misidentified lines (See ICLAC register)	No commonly misidentified cell lines were used in the study.
